# Our current understanding of the biological impact of endometrial cancer mtDNA genome mutations and their potential use as a biomarker

**DOI:** 10.3389/fonc.2024.1394699

**Published:** 2024-06-27

**Authors:** Pabitra Khadka, Carolyn K. J. Young, Ravi Sachidanandam, Laurent Brard, Matthew J. Young

**Affiliations:** ^1^ Department of Biomedical Sciences, Division of Biochemistry & Molecular Biology, Southern Illinois University School of Medicine, Carbondale, IL, United States; ^2^ Girihlet Inc, Oakland, CA, United States; ^3^ Obstetrics & Gynecology, Southern Illinois University School of Medicine, Springfield, IL, United States; ^4^ Simmons Cancer Institute, Springfield, IL, United States

**Keywords:** endometrial cancer (EC), mitochondrial DNA (mtDNA), heteroplasmy, homoplasmy, cancer biomarker, metabolism, endometrial cancer treatment, DNA polymerase gamma

## Abstract

Endometrial cancer (EC) is a devastating and common disease affecting women’s health. The NCI Surveillance, Epidemiology, and End Results Program predicted that there would be >66,000 new cases in the United States and >13,000 deaths from EC in 2023, and EC is the sixth most common cancer among women worldwide. Regulation of mitochondrial metabolism plays a role in tumorigenesis. In proliferating cancer cells, mitochondria provide the necessary building blocks for biosynthesis of amino acids, lipids, nucleotides, and glucose. One mechanism causing altered mitochondrial activity is mitochondrial DNA (mtDNA) mutation. The polyploid human mtDNA genome is a circular double-stranded molecule essential to vertebrate life that harbors genes critical for oxidative phosphorylation plus mitochondrial-derived peptide genes. Cancer cells display aerobic glycolysis, known as the Warburg effect, which arises from the needs of fast-dividing cells and is characterized by increased glucose uptake and conversion of glucose to lactate. Solid tumors often contain at least one mtDNA substitution. Furthermore, it is common for cancer cells to harbor mixtures of wild-type and mutant mtDNA genotypes, known as heteroplasmy. Considering the increase in cancer cell energy demand, the presence of functionally relevant carcinogenesis-inducing or environment-adapting mtDNA mutations in cancer seems plausible. We review 279 EC tumor-specific mtDNA single nucleotide variants from 111 individuals from different studies. Many transition mutations indicative of error-prone DNA polymerase γ replication and C to U deamination events were present. We examine the spectrum of mutations and their heteroplasmy and discuss the potential biological impact of recurrent, non-synonymous, insertion, and deletion mutations. Lastly, we explore current EC treatments, exploiting cancer cell mitochondria for therapy and the prospect of using mtDNA variants as an EC biomarker.

## Introduction

Endometrial carcinoma (EC) is the most common gynecologic malignant epithelial tumor type in the United States, with the death rate from this disease increasing by more than 100% over the past 20 years (1, 2). Worldwide, EC is the sixth most common cancer among women (3). According to recent estimates, there were ~90,000 deaths and ~382,000 new cases of EC in 2018 (4). In the United States, >66,000 new cases and >13,000 deaths from EC were predicted for 2023. Most EC deaths occur in middle-aged or older women, and uterine cancer is the fourteenth leading cause of cancer death. The age-adjusted death rate is estimated to be 5.1 per 100,000 women per year (5). EC is a devastating and common disease that results from the uncontrolled growth of cells within the endometrium, the inner layer or mucosal lining, of the mammalian uterus, which is comprised of an epithelial layer, glands, connective tissue (stroma), and blood vessels (6). Endometrial tissue is responsive to hormones, and the most common type of EC is thought to arise from estrogen stimulation that is unopposed by progestins.

Surgical staging provides vital information to predict the course of EC (i.e., prognostic information about how the cancer will affect an individual and respond to treatment). EC tumors are assigned an International Federation of Gynecology and Obstetrics (FIGO) histological grade based on the level of glandular differentiation. Grade 1, 2, and 3 tumors exhibit ≤5%, 6 to 50%, and >50% solid non-glandular, nonsquamous (non-flat cell) growth, respectively (7). Grade 1 and 2 tumors are referred to as “low-grade” tumors (more of the cells form glands), while grade 3 tumors are called “high-grade” (more of the cells do not form glands and are disorganized). Grade 3 tumors tend to be more aggressive and spread and grow fast. Women with early-stage/low-grade tumors have a more favorable prognosis compared to those with advanced disease/high-grade tumors (8).

ECs are generally classified into two types of tumors. Type I endometrioid adenocarcinoma tumors are the most common, representing more than 80% of EC cases, and are associated with unopposed estrogen stimulation. Interestingly, estrogen-mediated reactive oxygen species (ROS) production has been proposed to be a contributor to mitochondrial DNA (mtDNA) mutation (9). Type I ECs are generally low-grade tumors that exhibit glandular differentiation and likely originate from glandular cells, e.g., grades 1 and 2 endometrioid cancers (10). Type I ECs most often occur in obese post-menopausal women (and sometimes in anovulatory pre-menopausal individuals) and are associated with down-regulation or mutation of the *PTEN* tumor suppressor gene leading to protein kinase B (Akt) and mTOR (mammalian target of rapamycin, a phosphatidylinositol kinase-related kinase) activation (11). Type II tumors account for 10% of ECs and are associated with 40% of related deaths (12).

Type II ECs include heterogenous, undifferentiated carcinoma, carcinosarcoma (a mixture of carcinoma and sarcoma), serous carcinoma, clear cell carcinoma, and grade 3 endometrioid carcinoma. Type II ECs are less associated with estrogen stimulation and are typically poorly differentiated or high-grade tumors (13). Type II ECs are reported to be related to abnormal *TP53*, *HER2* (*ErbB2*), and *P16* and are often metastatic and associated with poor survival despite aggressive treatments with radiation and chemotherapy (11). A study by The Cancer Genome Atlas showed that most endometrioid tumors have few *TP53* mutations or copy number changes but frequent mutations in *KRAS*, *ARID1A*, *CTNNB1*, *PIK3CA*, and *PTEN* and novel mutations in the *ARID5B* gene. Uterine serous tumors and high-grade endometrioid tumors had extensive somatic changes to chromosome copy number and frequent *TP53* mutations, and a subset of endometrioid tumors had ultra-mutated *POLE*, encoding the catalytic subunit of DNA polymerase epsilon required for nuclear DNA replication and repair (14). Since the publication of the 2023 FIGO staging for endometrial cancer, molecular classification has been encouraged in all endometrial cancers. Molecular EC classification includes testing for pathogenic *POLE* mutation (*POLEmut*), mismatch repair deficient (MMRd) molecular subtypes, the non-specific molecular profile (NSMP) group, and *TP53* abnormal (p53abn) mutations (15, 16).

## The role of mitochondria (and mtDNA homoplasmy and heteroplasmy) in cancer

Mitochondria have several essential functions, such as steroid hormone biosynthesis, signaling, apoptosis, cell cycle control, production of energy, and so on (17, 18). Cells synthesize most of their ATP using the mitochondrial oxidative phosphorylation (OXPHOS) machinery, and this machinery requires 13 mtDNA-encoded proteins to function. Thus, proper mtDNA maintenance is essential to meet the basic energy demands within our cells. The multicopy mtDNA genome is replicated and repaired by the mtDNA polymerase gamma (Polγ) in concert with additional replisome factors, for example, Twinkle mtDNA helicase, topoisomerases, mitochondrial single-stranded DNA-binding protein, and others (19).

**Figure 1:**
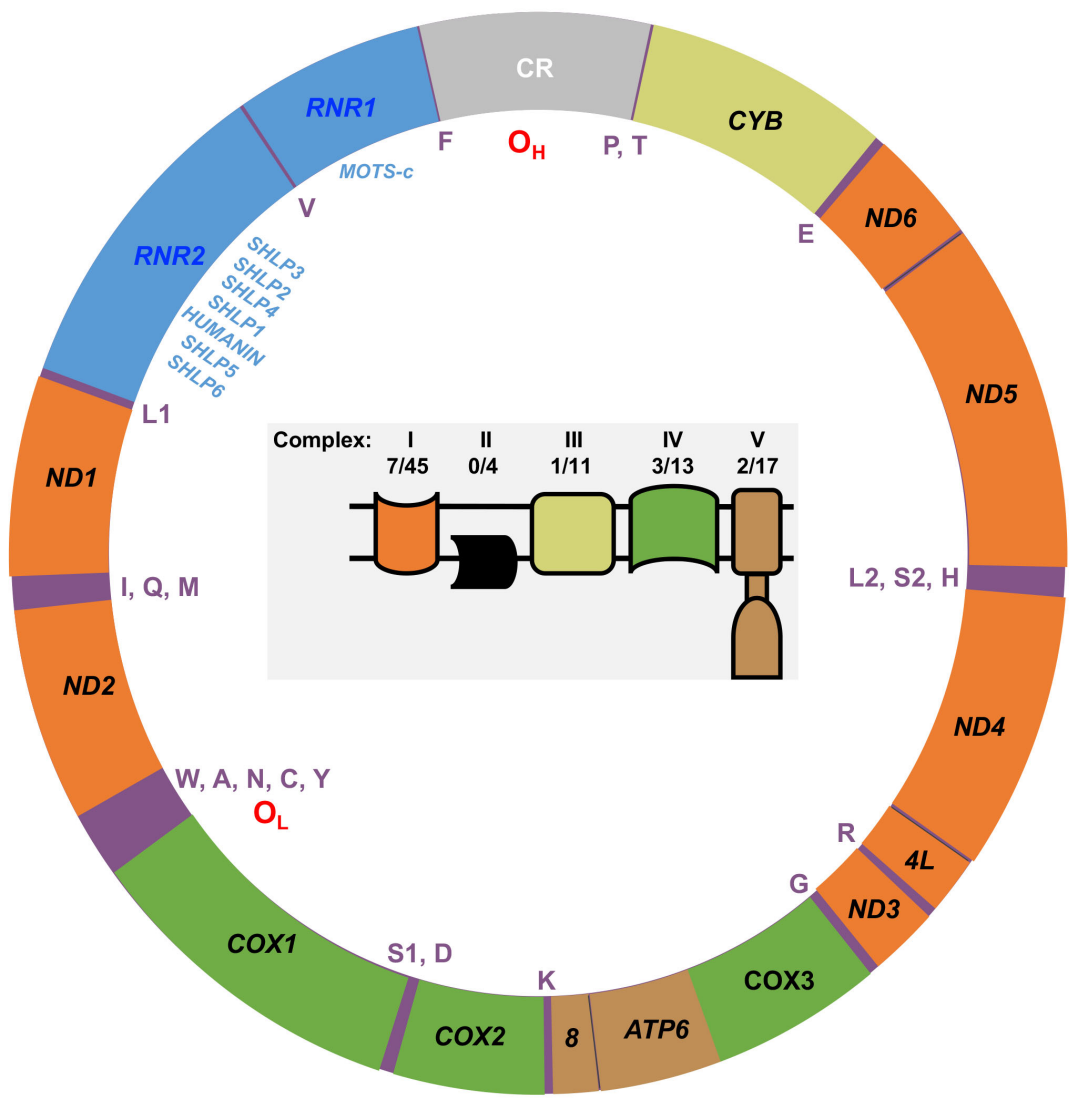
Map of the human mtDNA genome. The 13 genes encoding polypeptides of the mitochondrial OXPHOS machinery are labeled with black text on the map. Orange, OXPHOS complex I genes (NADH dehydrogenase, *ND1*, *ND2*, *ND3*, *ND4L*, *ND4*, *ND5*, and *ND6*); yellow, OXPHOS complex III gene (cytochrome bc1 complex cytochrome b, *CYB*); green, OXPHOS complex IV genes (cytochrome c oxidase, *COX1*, *COX2*, and *COX3*); brown, OXPHOS complex V genes (ATP synthase, *ATP6*, and *ATP8*). The small 12S (*RNR1*) and large 16S (*RNR2*) ribosomal RNA genes are colored blue, and the mtDNA control region (CR) is colored gray. The locations of the heavy strand origin of replication (O_H_), the light strand origin of replication (O_L_), the single letter amino acid residue codes for the 22 tRNA genes, and the eight mitochondrial-derived peptide (MDP) genes are indicated inside the map. The MDP genes localize within the *RNR1* and *RNR2* genes: HUMANIN, MOTS-c (Mitochondrial Open reading frame of The 12S rRNA-c), and the Small Humanin-Like Peptides 1 to 6 (SHLP1 – 6). Inside the mtDNA map is a cartoon of the mitochondrial inner membrane OXPHOS machinery. The OXPHOS complexes are color-coded according to their mtDNA gene colors on the map. Apart from complex II, the other OXPHOS complexes are encoded by both mtDNA and nDNA, and the number of mtDNA-encoded subunits out of the total number of complex subunits is indicated as previously reported (22).

The polyploid human mtDNA genome is a covalently closed circular double-stranded 16,569-bp molecule that harbors the 13 OXPHOS genes mentioned above in addition to 2 genes encoding rRNAs, 22 tRNA genes and 8 genes that code for the mitochondrial-derived peptide signaling molecules (MDPs) (20, 21), **Figure 1**. The 24 RNA genes are required to translate the 13 mtDNA-encoded OXPHOS polypeptides. The MDPs are bioactive peptides with various physiological functions. For example, MOTS-c (*M*itochondrial *O*pen reading frame of *T*he 12*S* rRNA-*c*) has been demonstrated to prevent diet-induced obesity and insulin resistance (23). MOTS-c is detectable in skeletal muscle and circulation and thus is described as a mitokine/mitochondrial hormone (24). MOTS-c translation occurs in the cytoplasm using the standard genetic code suggesting its RNA is exported from the mitochondria, while both mitochondrial and cytoplasmic expressed humanin have been suggested to be biologically active (20, 23).

Many cancers display aerobic glycolysis, also known as the Warburg effect (25). However, our current understanding is that tumor cells use both mitochondrial metabolism and increased glucose uptake and fermentation of glucose to lactate to synthesize ATP, macromolecules, and NADPH needed for rapidly proliferating cells (26, 27). In cancer cells, mitochondria provide the necessary building blocks for biosynthesis of amino acids, lipids, nucleotides, and glucose. During oncogenic activation, metabolism is rewired to generate ATP, and the needed citric acid cycle (CAC) intermediates utilized for macromolecule synthesis, e.g., citrate is exported from the mitochondria to the cytoplasm to generate the building blocks required for the biosynthesis of fatty acids and cholesterol (27–29).

**Figure 2:**
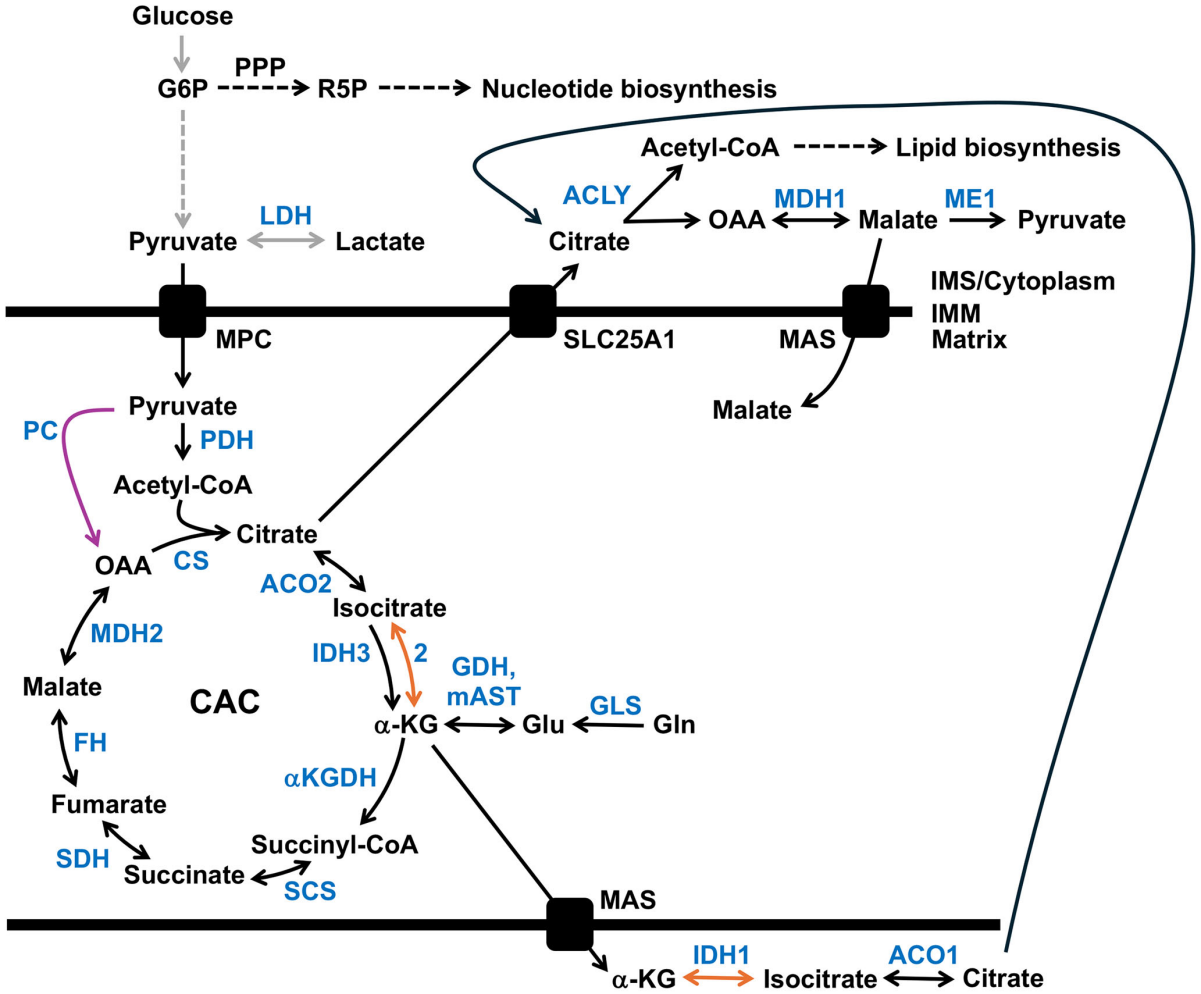
Metabolic adaptations in cancer. Gray arrows indicate the upregulation of glycolysis and enhanced lactate production, while the pyruvate carboxylase (PC) reaction is shown with a purple arrow. Reductive carboxylation of glutamine (Gln)-derived α-ketoglutarate (α-KG) to isocitrate is highlighted by orange arrows. Enzymes are highlighted in blue. See the text for details. ACLY, ATP citrate lyase; ACO1, cytosolic aconitase; ACO2, mitochondrial aconitase 2; αKGDH, α-ketoglutarate dehydrogenase complex; CAC, citric acid cycle; CS, citrate synthase; FH, fumarate hydratase (fumarase); G6P, glucose 6-phosphate; GDH, glutamate dehydrogenase; GLS, glutaminase; Glu, glutamate; IDH1, cytosolic isocitrate dehydrogenase; IDH2 and 3, mitochondrial isocitrate dehydrogenase 2 and 3; IMM, inner mitochondrial membrane; IMS, intermembrane space; LDH, lactate dehydrogenase; MAS, malate-aspartate (Asp) shuttle; mAST, mitochondrial aspartate aminotransferase; MDH1, cytosolic malate dehydrogenase 1 isozyme; MDH2, mitochondrial malate dehydrogenase; ME1, malic enzyme 1; MPC, mitochondrial pyruvate carrier; OAA, oxaloacetate; PDH, pyruvate dehydrogenase complex; PPP, the pentose phosphate pathway; R5P, ribose 5-phosphate; SCS, succinyl-CoA synthetase; SDH, succinate dehydrogenase complex; and SLC25A1, the dicarboxylate antiporter solute carrier family 25.

Metabolic characteristics of tumors arise from cell-intrinsic factors like metabolic phenotype of the cell of origin and transforming genetic lesions and environmental factors such as the nutrients available in the tissue microenvironments. Regarding mitochondrial rewiring in cancer, two anaplerotic mechanisms have been observed. Anaplerosis replenishes intermediates removed from the CAC that were used to supply other biosynthetic pathways. In xenografts of colorectal samples, glutamine catabolism generates α-ketoglutarate (α-KG), **Figure 2**. In contrast, pancreatic and lung tumors favor pyruvate carboxylation via pyruvate carboxylase (PC) to replenish mitochondrial oxaloacetate (29). In pancreatic cells, PC and malic enzyme (ME1) are predicted to participate in a pyruvate cycle whereby cytosolic ME1 generates NADPH and pyruvate from malate. Next, the cytosolic pyruvate can enter the mitochondria and be converted to oxaloacetate via mitochondrial PC (30).

Although most cancer cells harbor functional mitochondria, tumors with mutations impairing and altering mitochondrial metabolism have been identified. In cancer, the mutation of genes encoding mitochondrial proteins, including isocitrate dehydrogenase 1 and 2, succinate dehydrogenase, and fumarase, have been documented (9). In patient-derived UOK262 renal carcinoma cells with mutations in the fumarase/fumarate hydratase (*FH*) gene or cells with a mutation in an OXPHOS complex I subunit, a reductive, glutamine-dependent pathway is the primary metabolic pathway. Similar to UOK262 (*FH* defective), 143B cells engineered to contain a loss-of-function mtDNA mutation in the complex III *CYB* gene had higher levels of citrate, malate, and fatty acids produced by reductive carboxylation of glutamine-derived α-ketoglutarate. Furthermore, using RNA interference, the authors showed that silencing the *IDH1* (encoding the cytoplasmic isoform) or *IDH2* (mitochondrial isoform) genes but not *IDH3* (mitochondrial isoform) reduced the amount of glutamine-derived citrate implicating the IDH1 and IDH2 enzymes in the reverse reductive carboxylation reaction (31).

Could cancer cell heteroplasmic mutations in mtDNA similarly rewire metabolism? A recent study suggests yes and showed that murine cellular models of cancer-derived *ND5* mtDNA heteroplasmic mutations regulate cancer metabolism and tumor biology, with redox imbalance contributing to a Warburg-like metabolic shift to glycolysis. Cell lines were engineered to separately contain the G11944A (human G12539A/W68Term, ovarian serous cystadenocarcinoma) and G12436A (human G13031A/p.W232Term, kidney renal clear cell carcinoma) mtDNA heteroplasmic *ND5* termination mutations. Heteroplasmy-dependent increases in glucose-derived lactate and glutamine-derived cytoplasmic malate suggested the flow of reducing equivalents into the mitochondrion through the malate-aspartate shuttle (MAS) is impacted by changes in cellular redox. The increased abundance of glucose-derived lactate in heteroplasmic mutants was eliminated using the cyto*Lb*NOX system to increase the NAD+/NADH ratio (32). The cyto*Lb*NOX is a water-forming NADH oxidase genetic tool derived from *Lactobacillus brevis* that induces a cytosolic-specific increase of the NAD^+^ to NADH ratio in human cells. The human codon-optimized oxidase is specific for NADH rather than NADPH and catalyzes the reaction, 2NADH + 2H^+^ + O_2_ ➔ 2NAD^+^ + 2H_2_O (33).

Pyrimidine nucleotides play an essential role in metabolism, serving as RNA and DNA precursors, and uridine nucleotides act through receptors to regulate physiological processes (34). The *DHODH* gene is an essential gene that encodes the mitochondrial dihydroorotate dehydrogenase, an enzyme required for *de novo* pyrimidine base biosynthesis. The DHODH enzyme requires the functional activity of OXPHOS complex III (35, 36). The subunits of complex III are encoded by the *CYB* mtDNA gene and ten additional nuclear genes (37). As rapidly proliferating cancer cells have enhanced DNA replication and gene expression, glycolysis is likely increased to quickly generate ATP and intermediates needed by the pentose phosphate pathway to biosynthesize nucleotides. Augmented production of glycolytic ATP is predicted to provide an advantage to cancer cells as the rate of glycolytic glucose metabolism is 10-100 times faster than the complete oxidation of glucose through the mitochondria (26).

Furthermore, cancer cells display insensitivity to antigrowth signals, decreased autophagy, and impaired programmed cell death, i.e., apoptosis (28). In this model of rewired mitochondrial metabolism in cancer, mitochondria provide the essential TCA cycle intermediates needed by rapidly growing cells (38). Evidence for mitochondrial function in EC being critical for growth and proliferation comes from the observation that Type I (estrogen-dependent) patient samples demonstrate increased mitochondrial biogenesis compared to matched hyperplasia samples (39).

A cell can contain several thousand copies of circular mtDNA distributed within hundreds of individual mitochondria or throughout an elaborate mitochondrial reticular network (40). Human cancer cells harbor clonal synonymous (silent) and non-synonymous (non-silent, alters an encoded amino acid residue) mtDNA variants, so-called homoplasmic substitutions or homoplasmy. Additionally, cancer cells contain mixtures of wild-type (WT) and mutant mtDNA genotypes, known as mtDNA heteroplasmy (41–43). MtDNA mutation is a common feature of various types of cancer, and most solid tumors contain at least one mtDNA substitution (44). Mutations in mtDNA protein-coding genes can be silent or non-silent. Clinically critical variations in mtDNA can be categorized into three major classes: mtDNA germline mutations, somatic mutations, and ancient adaptive polymorphisms. Germline mutations are maternally inherited variants in the female germline; somatic mutations occur in oocytes, during embryogenesis, in somatic tissues, or in cancer tumors; mtDNA ancient adaptive polymorphisms result from selection during human migration (43).

## mtDNA maintenance is essential to vertebrate life

All multi-cellular organisms require mitochondria for bioenergetics and biosynthesis of precursors for macromolecules (45). In some organisms, such as humans, specific short-lived cells like red blood cells, which live for ~120 days, lack mitochondria (and nuclei), presumably to reduce their size and prevent them from using the oxygen they carry (46). The maintenance of mtDNA is essential to vertebrate life. Knockout (KO) of the mouse *POLG* gene encoding the catalytic subunit of Polγ revealed embryonic lethality at E7.5–8.5 with subsequent depletion of mtDNA (47). Comparatively, several studies have illustrated the essential role of the Polγ processivity subunit (or accessory subunit) p55 in mtDNA replication: (i) two separate null mutations in the *Drosophila melanogaster POLG2* gene lead to lethality in the early pupal stage of fly development (48), (ii) homozygous *POLG2* KO mice are embryonic lethal at E8–8.5 (49) and (iii) in a porcine oocyte knockdown model, oocyte maturation requires *POLG2* (50). Mouse RNaseH1*^-/-^* embryos are null at E8.5 and have decreased mtDNA content, leading to apoptotic cell death (51). A mouse model of Twinkle mtDNA helicase deficiency has been generated by transgenic expression of a Twinkle cDNA with an autosomal dominant mutation found in patients (52, 53). At one year of age, these mice developed progressive respiratory chain deficiency in cerebellar Purkinje cells, hippocampal neurons, and skeletal muscle. The affected cells accumulated multiple mtDNA deletions. These ‘Deletor’ mice recapitulate many of the symptoms associated with the *POLG*-related disease progressive external ophthalmoplegia and represent a valuable research model.

Our group recently generated another useful cell line research model of *POLG*-related mitochondrial disease. The human SJCRH30 myoblast cell line model harbors the most common autosomal dominant *POLG* mutation, c.2864A>G/p.Y955C, and displays bioenergetic deficits, decreased expression of OXPHOS complex I subunits, and impaired mtDNA maintenance (54). Furthermore, cancer cells lacking mtDNA fail to form tumors unless they reconstitute OXPHOS using mitochondria acquired from the host stroma (55). Therefore, mtDNA is essential to vertebrate biology and tumorigenesis.

## mtDNA heteroplasmic mutations in cancer

The Cancer Mitochondria Atlas (TCMA) surveyed 2536 high-quality matched cancer and control sample pairs from the Pan-Cancer Analysis of Whole Genomes Consortium covering 38 specific cancer types and identified 7611 somatic mtDNA substitutions and 930 small indels. Of the 7611 variants identified in the whole-genome sequencing (WGS) data, >85% were heteroplasmic. Additionally, in contrast with nuclear DNA (nDNA) mutations where cancer type-specific signatures are seen, mtDNA mutations are similar across different tumor types, and most of the mutations display strand bias with predominantly G > A and T > C substitutions on the L-strand (17).

A recent study utilized a sensitive whole-exome sequencing (WES) method focusing on tRNA, rRNA, and protein-coding genes. The study determined that predicted pathogenic mtDNA mutations arise in tumors at a rate similar to mutations in the standard cancer driver genes. However, it should be kept in mind that mtDNA mutations located in regions without adequate sequencing coverage are not identifiable with repurposed WES data, and this data can be biased towards variants with elevated heteroplasmy. In this study, 3,264 matched tumor and normal samples had sufficient coverage to call mutations in at least 90% of the mtDNA genome, and of these, fifty-seven percent harbored at least one mtDNA variant. Interestingly, predicted pathogenic mtDNA mutations were shown to be associated with increases in the survival of colorectal cancer patients. The *CYB* cytochrome b gene showed increased rates of missense mutations, complex I genes were shown to accumulate loss-of-function mutations at homopolymeric runs at an increased rate, and complex V genes were depleted of non-synonymous mutations, implying negative selection against ATP synthase gene mutations. Also, the transcriptional analysis from this study showed truncating mtDNA mutations promote decreased expression of innate immunity genes and increased expression of OXPHOS genes (56).

To identify somatic mutations in cancer, we assume a particular mutation present in the cancer tissue will be absent in the normal tissue of the same individual (17, 57). However, the effect of most of these somatic mtDNA mutations and their exact role in carcinogenesis remains unclear. MtDNA mutations have been found to hinder energy production, cause metabolic disorders, and alter the production of ROS (58). The rate of mutation in mtDNA genes is estimated to be 100- to 1000-fold higher than nDNA genes, which makes mtDNA a good candidate for involvement in cancer progression (59, 60). It is reasonable to conceive that cancer mtDNA mutations can be either driver mutations, providing a selective growth advantage to a cell, or passenger mutations, not providing any advantage. Considering the increase in cancer cell energy demand compared to normal cells, functionally relevant driver mtDNA mutations in cancer seem plausible (61). Furthermore, mtDNA *de novo* mutations are hypothesized to function as either strong ‘inducers’ of carcinogenesis or mild ‘adaptors’ that permit a cancer cell to adapt to different environments (43). As the mtDNA and nDNA genomes encode subunits of the OXPHOS machinery, mutations in either genome could alter ROS production or the cell’s redox status, contributing to tumor growth (62). Changes in mitochondrial genes encoded on either genome could alter mitochondrial metabolites, further altering gene expression and contributing to tumor growth (43).

Further evidence demonstrating the essentiality of vertebrate mtDNA comes from the hundreds of documented pathogenic mtDNA mutations linked with multisystem degenerative disorders (60). Therefore, in cancer, it has been hypothesized that neutral missense mutations drift toward homoplasmy while deleterious or pathogenic mutations are under negative selection to remove harmful mutations (63). Suppose cancer cell mtDNA mutations are predicted to be detrimental if homoplasmic; why are they not removed by purifying (negative) selection, and is there some selective advantage to a cancer cell maintaining a predicted pathogenic mtDNA mutation in heteroplasmy? In cancer cells, we expect that mitochondria are under selective pressure to keep the mtDNA-encoded OXPHOS subunits. Therefore, if a mtDNA gene is mutated to alter the function of its encoded protein detrimentally, then the organelle can compensate by maintaining a mixed population of WT and mutant mtDNA molecules (57). We hypothesize that EC cells benefit from carcinogenesis-inducing or environment-adapting heteroplasmic somatic mtDNA mutations (predicted pathogenic) that favor EC metabolism, hereafter inducing/adapting mutations. On the other hand, we expect more mild (less damaging or pathogenic) environment-adapting mutations could drift towards mtDNA homoplasmy.

## EC-specific mutations occur across the mtDNA genome

Next, to understand EC mtDNA mutations that occur across the entire mtDNA genome, we review mtDNA data obtained from Mseek and WGS studies. The tumor-specific mtDNA mutations, heteroplasmy levels, and the mutations’ predicted impacts are discussed below. Previously, we used the Mseek mtDNA-specific next-generation sequencing (NGS) approach to investigate the entire mtDNA from 3 matched sample sets (tumor and peri-normal) and identified nine single nucleotide somatic EC mtDNA variants. Mseek exploits mtDNA circular topology and treatment of DNA samples with exonuclease V to reduce the amount of nuclear genomic reads. The coverage of our Mseek data was >400x (57). One hundred and ninety-one somatic EC mtDNA variants from 102 sample sets were obtained from the NGS study by Grandhi et al. that utilized data accessed from The Cancer Genome Atlas (TCGA) August 2015. Tumor and matched normal sample sets were compared to determine an appropriate uniform read depth to detect somatic mutations, >50x (64). One hundred forty-five variants from 40 samples were obtained from the ultra-high depth Yuan et al. NGS study, with >5,000x average coverage for uterus adenocarcinoma samples. The group extracted mtDNA sequence data from the whole-genome alignment files of cancer samples and their matched normal tissue samples obtained from The Pan-Cancer Analysis of Whole Genomes (PCAWG) Consortium. The PCAWG aggregated WGS data from the International Cancer Genome Consortium (ICGC) and TCGA projects (17).

**Figure 3:**
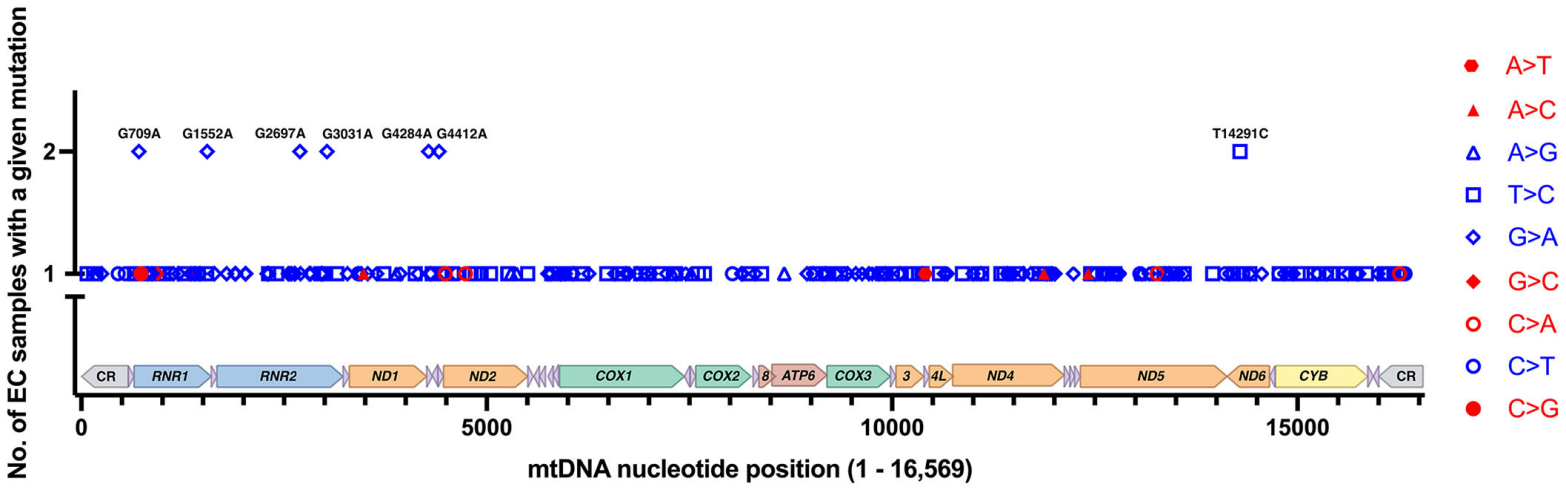
EC-specific mutations and their positions on a linear map of the mtDNA genome. Of the 279 mutations from 111 samples, 272 are unique, and 7 reoccurred in separate samples. Transitions are shown in blue, and transversions are in red. Each type of mutation is represented with a different symbol. The mutations shown on the graph are changes from the rCRS NC_012920.1. The mtDNA genes are colored as in **Figure 1**.

The Grandhi et al. and Yuan et al. studies both mined data from TCGA, but at different points in time and using different bioinformatics methods to call mtDNA variants and heteroplasmy. Therefore, we carefully scrutinized the results to remove likely duplicated samples with identical mutations and highly similar levels of heteroplasmy. On average, the duplicates had a difference of 2.1% heteroplasmy. Based on the duplicate analysis, we removed 34 samples with 66 mutations from the set obtained from the Grandhi et al. study, and the matched mutations in the Yuan et al. study were kept in the analysis. The result is 279 EC tumor-specific single nucleotide variants from 111 samples (an average of 2.5 variants per sample), **Supplementary Table 1** (17, 57, 64). Seven recurrent mutations were identified within this set of variants, three from the Grandhi et al. and four from the Yuan et al. studies, **Figure 3**.

According to the strand displacement model of mtDNA replication, replisomes containing Polγ synthesize both the nascent heavy (H) and light (L) strands continuously without the formation of Okazaki-fragment-like replication products (65). The two mtDNA strands are named H and L based on the ability to separate them on denaturing cesium chloride gradients. The H-strand is richer in G+T content, making it heavier on density centrifugation (66, 67). The origin of H-strand DNA replication (O_H_) is located in the non-coding control region (CR), and the origin of L-strand replication (O_L_) is located ~11,000 base pairs downstream of O_H_, **Figure 1** (65). The CR harbors a three-stranded DNA zone known as the displacement loop or D-loop (68, 69). Another difference from nDNA replication is that mtDNA molecules are replicated independently of the cell cycle (70). During mtDNA replication, relatively long single-stranded stretches of the polyploid H-strand templates are formed, which can be argued to be sensitive targets of exogenous and endogenous damage and need protection during the replication process by the mitochondrial single-stranded DNA binding protein, mtSSB (54).

The EC-specific mtDNA variants represent changes from the revised Cambridge Reference Sequence (rCRS). Out of the 279 EC tumor-specific mtDNA mutations, transitions were 24.4-fold higher than transversions (268 transitions and 11 transversions), **Figure 3**. The high transition: transversion ratio (ts/tv) has been previously noted in human mtDNA, with transitions being 15-fold higher than transversions (71); however, in EC tumors, the ratio is even higher. EC-specific mutations occurred across the mtDNA genome in both non-coding regions (CR, NC5, and O_L_) and coding genes except for ten tRNA genes (TRNF, Q, W, A, N, Y, H, L2, E, and T), which did not harbor any mutations. Of the total mutations, 65.9% occur in protein-coding genes, 7.5% in tRNA genes, 20.4% in rRNA genes, and 6.1% in the non-coding regions. The mutations in protein-coding genes were both synonymous and non-synonymous. About one-quarter of the 57 mutations in the rRNA genes localize to the MDP open reading frames. These consisted of a synonymous *MOTS-c* mutation, three silent mutations in *RNR2* MDP genes (*SHLP5*, *SHLP6*), and ten non-synonymous mutations in *RNR2* MDP genes (*SHLP3*, *SHLP1*, *Humanin*, *SHLP5*, and *SHLP6*).

## EC mtDNA mutations reoccur

Seven EC-specific mtDNA transition mutations reoccurred in more than one sample, and these comprised 14 of the 279 mutations, G709A (*RNR1*), G1552A (*RNR1*), G2697A/R22K (*Humanin*/*RNR2*), G3031A/K14K (*SHLP6*/*RNR2*), G4284A (*TRNI*), G4412A (*TRNM*), and T14291C/E128G (*ND6*), **Figure 3**. Eleven of the 14 recurrent mutations fell between 10 and 90% heteroplasmy except for one of each of G2697A (90.02% heteroplasmy), G3031A (96.74%), and T14291C (3.95%), **Supplementary Table 1**. Unfortunately, there is currently no sufficient model to predict a mutation’s pathogenicity when it occurs in the mtDNA rRNA genes (*RNR1*, *RNR2*), the short open reading frame MDP signaling molecules (*humanin*, *MOTS-c*, and *SHLP1 – 6*), or the non-coding regions (CR, NC5, and O_L_). The MITOMASTER mtDNA sequence analysis tool (72) revealed that apart from the frequent G709A variant (present in the three L/African, M/Asian, and N/Eurasian human mtDNA Lineages in Mitomap at 11.32%, 9.87%, and 14.31% respectively), the remaining recurrent variants were present at 0% or equal to or less than 0.007% in the GeneBank, gnomAD (73), and HelixMTdb (74) reference population databases. Furthermore, a significant amount of the total 279 variants were 0%, or there was “No Record” in the GeneBank, gnomAD, and HelixMTdb databases, 56% (156 variants), 63% (176 variants), and 58% (161 variants), respectively. However, care needs to be taken when assessing variants as a rarity in the population should not be considered the only line of evidence for a mutation’s pathogenicity as many unrepresented variations are benign and databases are far from saturation (73).

Using the Mitochondrial tRNA Informatics Predictor (MitoTIP) *in silico* tool (75), the *TRNI* G4284A mutation is predicted to be possibly benign but is associated with spastic paraparesis (76). The *TRNM* G4412A mutation is expected to be likely-pathogenic and is associated with seizures, myopathy, and retinopathy (77); therefore, when present in heteroplasmy at 27.63 or 66.85%, the G4412A tRNA mutation is predicted to be an inducing/adapting mutation that alters EC cellular metabolism. The silent *SHLP6* G3031A/K14K mutation is expected to be non-harmful, but as mentioned previously, the effect of the mutation on *RNR2* cannot currently be predicted.

Non-synonymous mutations were categorized into predicted inducing/adapting mutations (predicted pathogenic) and non-pathogenic groups. Utilizing the MitImpact database collection of genomic, clinical, and functional annotations for non-synonymous mutations in mtDNA protein-coding genes (78), we scored a mutation as a predicted inducing/adapting mutation that is potentially able to favor EC cell metabolism if at least two out of three pathogenicity predictor scores were deleterious [CAROL, Combined Annotation scoRing tool (79)], likely-pathogenic/pathogenic [APOGEE2, pAthogenicity Prediction thrOugh loGistic modEl trEe (80)], and medium/high-impact [Mutation Accessor (81)]. Although the T14291C/E128G (*ND6*) variant is absent from the GeneBank, gnomAD, and HelixMTdb databases, the *in-silico* pathogenicity scores predict it is likely non-pathogenic. Of the variants shared among two patients described above, four occurred in the rRNA genes. Also, two *RNR2* gene mutations encode mutations in MDP genes, one is non-synonymous (*Humanin* G2697A/R22K), and the other is silent (*SHLP6* G3031A/K14K).

Thus, out of the 279 EC mtDNA mutations identified in 111 patients, 265 occurred in single samples (do not reoccur), and 272 unique mutations were identified in the cohort. Of the 265 non-reoccurring variants, 49 localize in rRNA genes and 17 in non-coding regions. For the remaining non-reoccurring variants, 83 are predicted to be inducing/adapting mutations (termination mutations, non-synonymous mutations, and pathogenic tRNA changes), 61 are non-synonymous non-pathogenic, 8 are tRNA non-pathogenic mutations, and 47 are silent substitutions. Next, we consider the 279 mutations in aggregate to gain insight into the current collection of tumor-specific mtDNA mutations identified in the EC cohort.

## mtDNA transitions are the most abundant mutations in EC tumors

**Figure 4:**
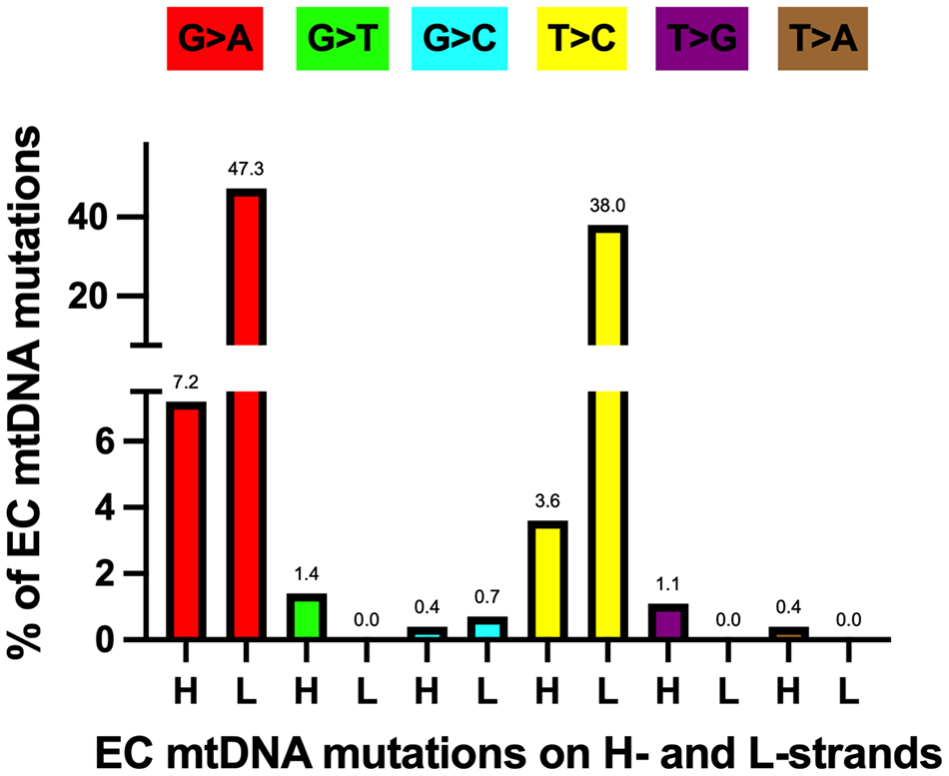
Most EC-specific G>A and T>C transition mutations occur on the mtDNA light (L) strand. H, heavy strand. Each mutation is shown as a percentage of the total mutations. Different colored bars represent each substitution type; the mutation type is shown above each bar. Based on data from references (17, 57, 64), see **Supplementary Table 1**.

As mentioned above, EC tumor-specific transition mutations were 24.4-fold higher than transversions. The increased level of EC mtDNA transitions agrees with a study of 1907 mtDNA mutations from 31 different cancer types that found transitions represent most of the substitutions (61). Furthermore, Ju et al. found strand bias concerning these transition mutations, with 76.8% of the T>C mutations on the L-strand (T_L_:A_H_ to C_L_:G_H_) and 84.1% of the C>T variants on the H-strand (G_L_:C_H_ to A_L_:T_H_). The strand bias is present in the EC samples, with 91.4% of the T>C mutations on the L-strand and 86.8% of the C>T variants on the H-strand. Transition mutations were the most abundant changes in the EC tumor mtDNAs when normalized to all other mutations, with 47.3% G_L_:C_H_ to A_L_:T_H_ and 38.0% T_L_:A_H_ to C_L_:G_H_, **Figure 4**. These transitions likely arose from 1. H-strand C to U deamination events and 2. erroneous incorporation of a nascent H-strand G across from a L-strand template T by the replicative Polγ followed by subsequent rounds of mtDNA replication resulting in C_H_>T_H_ and A_H_>G_H_, respectively, and as previously suggested (61, 82, 83). We propose the single-stranded nature of replicating mtDNA drives the accumulation of C_H_>T_H_ mutations in cancer. This is because C>T transitions resulting from spontaneous deamination of C to U have been shown to occur at orders of magnitude more quickly in ssDNA than dsDNA (84), and because replication intermediates harbor significant stretches of H-strand ss-mtDNA. Furthermore, because Polγ is prone to inserting G across from a template T, and as estrogen stimulates the biogenesis of mitochondria (9), the high levels of EC mtDNA A_H_>G_H_ mutations likely represent signatures of increased mtDNA replication in cancer.

The subsequent most abundant mutations were 7.2% G_H_:C_L_ to A_H_:T_L_ and 3.6% T_H_:A_L_ to C_H_:G_L_ and these could have arisen at short single-stranded regions of the replication fork by similar means as described above but on the opposite strand. EC tumor transversion mutations were low, with G_H_:C_L_ to T_H_:A_L_ (1.4%) and T_H_:A_L_ to G_H_:C_L_ (1.1%) being the most abundant. Due to the proximity of the mtDNA nucleoid to the OXPHOS machinery, we hypothesize that the G_H_>T_H_ mutations result from ROS-induced deoxyguanosine base damage in the form of 7,8-dihydro-8-oxo-2’-deoxyguanosine, 8-oxo-dG (85). In this scenario, Polγ would mis-incorporate an A across from the H-strand template containing the 8-oxo-dG base, resulting in a G>T transversion following mtDNA replication. Finally, T_L_:A_H_ to A_L_:T_H_, T_L_:A_H_ to G_L_:C_H_, and G_L_:C_H_ to T_L_:A_H_ transversions were not detected in the EC tumors.

## Half of the tumor samples harbor mtDNA mutations predicted to favor EC metabolism

**Figure 5:**
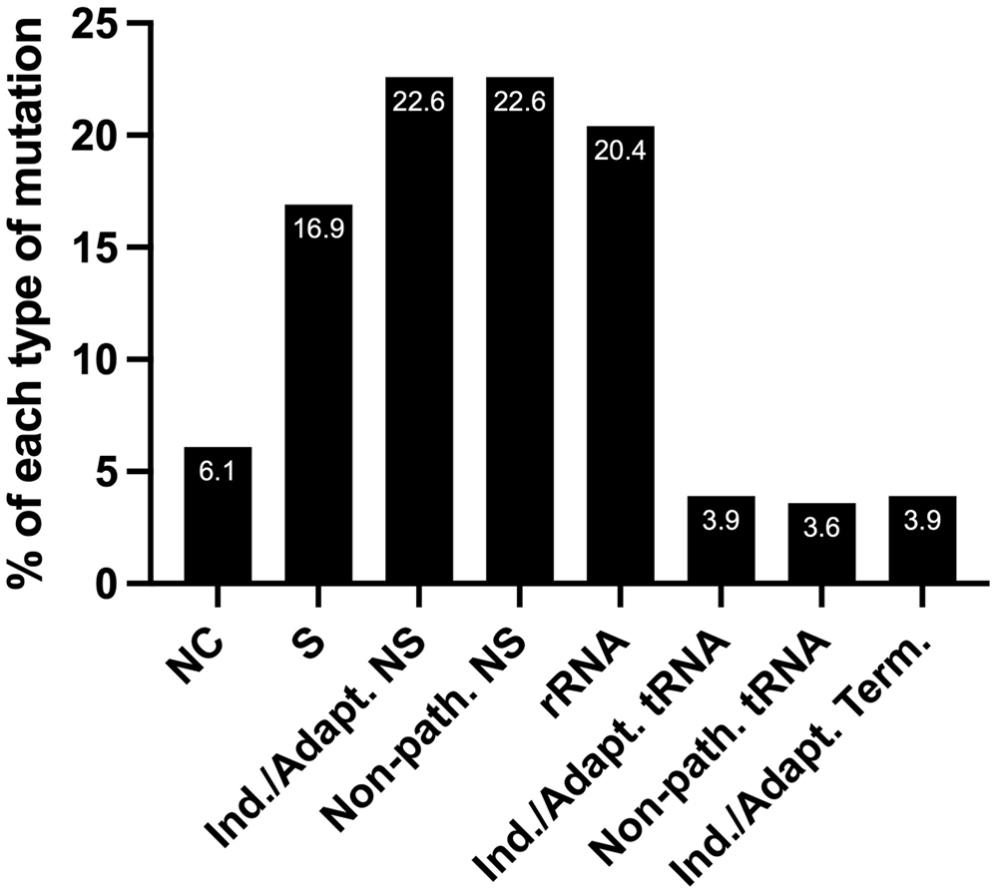
The percentage of each type of mutation from the set of 279 EC mtDNA somatic substitutions. NC, non-coding; S, synonymous (silent); NS, non-synonymous (non-silent). Nearly a third of the substitutions are predicted carcinogenesis-inducing or environment-adapting mutations that favor EC metabolism (Ind./Adapt.); Non-path., predicted non-pathogenic; Term., termination; see the text for details.

We predict that out of 279 mtDNA substitutions, 63 are non-synonymous inducing/adapting mutations (22.6%), and another 22.6% are non-synonymous and non-pathogenic, **Figure 5**. Eleven substitutions occurring in tRNA genes are predicted to be inducing/adapting mutations (3.9%), and 10 are likely non-pathogenic (3.6%). There are 11 mutations in the protein-coding genes that formed termination or nonsense codons (3.9%), and of these, 10 are G_L_:C_H_>A_L_:T_H_. We expect that the mtDNA termination mutations are inducing/adapting mutations that can favor EC metabolism as they are predicted to alter their respective protein’s function. Utilizing the MitImpact database, we determined the impact of changing the amino acid residues removed because of the various termination codons. In all these cases, changing the amino acid residues at the termination positions is predicted to alter metabolism, as the other two MitImpact SNVs considered at these positions were predicted to be impactful, deleterious, and pathogenic with at least two out of three pathogenicity predictor scores as described above. Moreover, changing a sense codon to a stop codon can lead to the loss of many amino acids and the complete protein will not be formed. In our analysis, these nonsense mutations truncated 11.6% to 99% of the proteins, with an average of 55% being truncated. The lost amino acid residues are likely essential to the function of their respective protein. Six of the 11 unique nonsense mutations localize to complex I genes (one mutation in *ND4* and five in *ND5*), which agrees with the previous observation that truncation mutations preferentially arise in complex I genes relative to other genes (56).

Defective complex I in cancer has been proposed to potentiate metastasis by enhancing ROS production, promoting tumor formation, and increasing resistance to cell death stimuli. Additionally, *ND5* gene mutations are linked to different cancers like colon adenocarcinoma, acute myeloid leukemia, breast cancer, and myelodysplastic syndrome (a.k.a. MDS, preleukemia), and these changes are suggested to inhibit OXPHOS resulting in altered mitochondrial bioenergetics which could confer a selective growth advantage to cancer cells (86). In terms of the 111 EC tumors with 279 tumor-specific mutations, 107 mutations occur in complex I genes, and the majority occur in *ND5* (11 in *ND1*, 14 in *ND2*, 9 in *ND3*, 24 in *ND4*, 3 in *ND4L*, 36 in *ND5*, and 10 in *ND6*). Of these variants, six result in termination mutations, 28 are silent, and 73 are non-silent. Thirty-three of the non-silent mutations are predicted to be inducing/adapting mutations, and 40 are expected to be non-pathogenic.

For 279 mutations, heteroplasmy ranged from 1 to 98.8%, averaging 49.2%. As we hypothesize that tumors could benefit from heteroplasmic mtDNA inducing/adapting mutations that favor EC metabolism, we looked at tumors that harbored at least one mtDNA mutation allele at 10% (WT 90%) to 90% (WT 10%) heteroplasmy (assuming slight to significant changes in EC tumor metabolism, respectively). We found that 79.3% of the 111 tumor samples contained at least one heteroplasmic mutation within this range, **Supplementary Table 1**.

Next, we wondered how many patients harbored predicted inducing/adapting mutations because they represented nearly a third of the total mutations (22.6 + 3.9 + 3.9%), **Figure 5**. Interestingly, half of the patient samples (56/111) harbored a predicted inducing/adapting mutation, which is significant considering it is currently not possible to predict the effects of a similar proportion of rRNA (20.4%) and non-coding (6.1%) mutations. The remaining 16.9% of the EC mtDNA substitutions are silent variants, **Figure 5**.

Of the 85 predicted inducing/adapting mutations, 46 have heteroplasmy levels between 10 and 90% (including 7 of 11 termination mutations and 9 of 11 tRNA mutations). Twenty-three non-synonymous predicted inducing/adapting mutations were <10% heteroplasmy, while 16 had >90% heteroplasmy (2 tRNA and 14 non-synonymous mutations in protein-coding genes). We expect the 16 with greater than 90% heteroplasmy could be mild environment-adapting mutations drifting towards homoplasmy. On the other hand, we speculate that the 23 non-synonymous predicted inducing/adapting mutations with less than 10% heteroplasmy may have been isolated from low-grade tumors or tumors surgically removed earlier during carcinogenesis. There were 3 predicted inducing/adapting termination mutations with less than 10% heteroplasmy, and 1 with greater than 90% heteroplasmy, and 2 tRNA inducing/adapting mutations with >90% heteroplasmy, **Supplementary Table 1**.

The 85 predicted inducing/adapting somatic mtDNA mutations exist among 56 tumor samples, suggesting there could be synergistic effects on metabolism when different mutations are in the same tumor. Approximately 59% of the tumor samples (33 of 56) harbored at least one predicted inducing/adapting mutation that falls between 10 and 90% heteroplasmy (12 tumors harbored predicted inducing/adapting mutations at <10% heteroplasmy and 11 had mutations with >90% heteroplasmy). Thirteen of the tumor samples harboring predicted inducing/adapting mtDNA mutations outside the 10 to 90% range also had mutations in either the rRNA genes or the non-coding regions. Therefore, 30% of the total 111 tumor samples harbor a predicted inducing/adapting somatic mtDNA mutation between 10 and 90% heteroplasmy, suggesting these variants could play an essential role in EC metabolism.

## EC mtDNA insertions and deletions reoccur in different samples

Twenty-eight mtDNA indels (16 insertions and 12 deletions) in 25 EC samples were identified, **Supplementary Table 2**. Eighteen of the samples with indels also had mtDNA somatic mutations (i.e., 18 of 25 samples with indels were also part of the 111-sample cohort with 279 total somatic mutations). Heteroplasmy was not reported for 18 of 28 indels, but the remaining 10 had heteroplasmy ranging from 16 to 95%, with an average of 52%. Approximately 68% of the indels occurred in coding regions (19 mutations), and ~54% occurred specifically in complex I genes. 79% of the indels were single nucleotide insertions or deletions. Although the pathogenicity predictor tools do not predict the impact of indels, we hypothesize that frameshift mutations in the coding region of a mtDNA-encoded protein will be inducing/adapting mutations that alter the protein’s function and, by extension, the cell’s metabolism. The three nucleotide in-frame deletion TAGC12989T does not disrupt the reading frame but does delete the evolutionarily conserved A219 amino acid residue of ND5. Six indels occurred in non-coding regions (4 in the CR, 1 in NC5, 1 in NC7), one in *RNR2*, and two in tRNA genes, *TRNS1* and *TRNP*.

Five EC mtDNA indels in complex I genes reoccurred in more than one sample, comprising 11 of 28 indels (39%). Of the five reoccurring indels, the *ND4* A11866AC insertion is shared among three individuals, while the remaining variants are shared among two individuals, *ND1* A3565AC, *ND4* A10946AC, *ND5* CA12417C, and *ND5* C12417CA. Interestingly, more than half of the reoccurring mtDNA indels, A11866AC (m.11872insC), CA12417C (m.12425delA), and A3565AC (m.3571insC), have been reported to likely induce oncocytoma (43). Therefore, these mutations may also be inducing/adapting mutations that favor EC metabolism and could serve as biomarkers for EC detection. Of the 28 EC mtDNA indels identified in 25 samples, 17 do not reoccur, and 22 unique indels were identified in this cohort.

## mtDNA copy number changes in EC

Human mtDNA occurs at a high copy number and varies between cell types, ranging from >150,000 to ~100,000 copies in mature oocytes, 1000s of copies per muscle fiber, and an average of 100 copies per sperm cell (87–89). There are interindividual variabilities in mtDNA copy numbers in human cells, which have been suggested to be linked to pathological features like obesity and cancer (90). Studies have shown that a high mtDNA copy number in whole-blood DNA extracts is linked to lung and breast cancer risk (91, 92). In contrast, low mtDNA copy number in whole-blood DNA extracts is associated with risks of renal cell carcinoma, soft tissue sarcoma, and esophageal adenocarcinoma (93–95). Another study showed that mtDNA from whole-blood DNA extracts is present at lower levels than in controls and is associated with an increased risk of endometrial cancer (90). Additionally, mtDNA content increased in hyperplastic and EC tissues compared to control tissue (96). In different studies with n ≥32 control samples and n ≥40 EC samples, 2- to 2.6-fold increases in mtDNA content were found in EC tumors compared to normal tissue (97, 98). In a study carried out by our group looking at three EC patients’ matched tumor and peri-normal tissues, we found that mtDNA copy number was increased relative to peri-normal tissue in one patient, decreased in another, and did not change in the third individual. In the same patients, mtDNA topological isomers (topoisomers) were studied, and catenated species were increased in the three EC tumor samples relative to the matched peri-normal specimens, suggesting enhanced mtDNA replication in the tumors (57). Interindividual variabilities in mtDNA could explain the lack of increased mtDNA copy number levels in particular EC samples in the various studies.

## mtDNA haplogroups and EC

Substantial mtDNA sequence diversity exists between individuals and human populations. Evidence suggests that ancient mtDNA polymorphisms accumulated along maternal lineages as humans migrated out of Africa. If a mutation changed mitochondrial physiology to benefit individuals within that environment, then that variant became enriched in that location. Further mutations in descendant mtDNA genomes generated a group of related regional mtDNA variant genotypes known as haplogroups. Therefore, each continent and geographical region is associated with characteristic mtDNA haplogroups (60). Haplogroups A, B, C, D, and E are specific to Asian populations, and one study investigated the relationship between EC and mtDNA variation in subjects with a Han native background in southwestern China. Using Chi-square statistics, frequency differences of haplogroups were tested between an EC group (n = 49 cancer patients) and a control group (31 control individuals). The study found that EC patients clustered in haplogroup D with a significantly higher frequency than controls (χ^2^ = 5.685, P = 0.017), suggesting a possible association of haplogroup D to EC. Additionally, the mtDNA haplogroup D C5178A allele frequency between EC patients and control subjects was significant (χ^2^ = 7.143, P = 0.007), suggesting it could be linked to EC pathogenesis (99).

Haplogroups H, I, J, K, T, U, V, W, and X are distributed among the European populations. In a study conducted in Lublin, Poland, mtDNA sequences of 26 EC patients were compared with the general Polish population to test for an association with cancer susceptibility. The Haplogroup H C7028T polymorphism was strongly underrepresented (χ^2^ = 8.58, P = 0.003) in three EC patients relative to the general Polish population suggesting haplogroup H could be a cancer-protective group (100). Future haplogroup studies with significant numbers of EC patients and general population mtDNA sequence data are needed to understand better how regional populations and their haplogroups are at risk of developing EC.

## Treatment of EC

Common treatments for cancer include surgery, radiation therapy (RT), and chemotherapy. Standard EC treatment involves surgery, i.e., the removal of the uterus, cervix, fallopian tubes, and ovaries, and selective pelvic and para-aortic lymphadenectomy. Women who are not candidates for surgery can be recommended RT. Additionally, progestin-containing intrauterine devices (IUDs) can be used as an alternative for young individuals wishing to preserve fertility, as these devices have been demonstrated to regress endometrioid EC (101–103). Following treatment, patients may undergo hormonal therapy, RT (external beam and/or vaginal brachytherapy), or chemotherapy, depending on their risk factors and stage of disease. Hormonal therapy has been primarily evaluated in low-grade endometrioid cancers. Agents include Megace (or megestrol, a progestin of the 17α-hydroxyprogesterone group) alternating with tamoxifen, progestational agents, and aromatase inhibitors. Good responses have been seen in patients with ER/PR-positive disease, low-grade disseminated disease, and pulmonary metastases (104–112). Chemotherapy treatment for metastatic EC can utilize single or multiple agents, including taxanes (e.g., paclitaxel), anthracyclines (e.g., doxorubicin), and platinum compounds (e.g., cisplatin) (11). These treatment options are highly individualized (and beyond the scope of this review).

First-line therapies have moved beyond paclitaxel and carboplatin (another platinum-containing compound) and now include immunotherapy agents in combination with chemotherapy. Humanized antibody pembrolizumab and monoclonal antibody dostarlimab are approved immune checkpoint inhibitors used in treating patients with recurrent or primary advanced EC that are either deficient (MMRd) or proficient (MMRp) in DNA mismatch repair. During nDNA synthesis, the incorporation of an incorrect nucleotide sometimes occurs, and cells use MMR protein machinery to repair these errors. Because MMRd EC tumors produce abnormal variant proteins, they tend to attract immune cells. Two recent large, randomized trials, NRG-GY018 (113) and RUBY (3) have shown a progression-free survival benefit by adding pembrolizumab (NRG-GY018) or dostarlimab (RUBY) to paclitaxel and carboplatin in patients with stage III or IVA endometrial carcinoma with measurable disease, or stage IVB or recurrent disease of any histologic subtype, (except for carcinosarcoma for pembrolizumab). Significantly, patients with either MMRd or MMRp benefited from these combinations, with the greatest benefit seen in patients with MMRd tumors.

## Exploiting cancer cell mitochondria for cancer therapy

Mitochondria are increasingly considered targets for cancer therapy due to their essential role in programmed cell death (i.e., apoptosis), cellular metabolism, and cell signaling (114). Researchers have focused on designing compounds that function as mitochondrial-targeting ligands and carry anticancer agents to the organelle. Examples of mitochondrial-targeting ligands include triphenylphosphonium (TPP, a cationic molecule that penetrates through mitochondrial membranes and accumulates in mitochondria) attached to the anticancer DNA damaging agent chlorambucil (chlorambucil linked TPP) and cationic mitochondria-penetrating peptides (MPPs) attached to anticancer drugs (e.g., doxorubicin linked MPP) (28). Cancer therapies that target mitochondria can inhibit energy supply to kill cells. Also, compounds that interfere with the electron transport chain, a significant site of ROS production, can increase the risk of electron leakage and ROS production, which can then damage the mitochondria and lead to apoptosis.

Mitochondria also play an essential role in ferroptosis, an iron-dependent form of non-apoptotic cell death driven by lipid peroxidation due to iron accumulation. Like apoptosis, ferroptosis induces decreased mitochondrial membrane potential but, unlike apoptosis, does not require caspase activation. In a study using HepG2 and Hep3B liver cancer cell lines, the CDGSH iron sulfur domain 1 iron-containing outer mitochondrial membrane protein (CISD1, also named mitoNEET) was shown to inhibit ferroptosis by protecting against mitochondrial lipid peroxidation. CISD1 is an iron-sulfur (2Fe-2S) protein that regulates iron transport into the mitochondrion. Stabilizing the CISD1 2Fe-2S cluster by pioglitazone inhibited mitochondrial iron import, lipid peroxidation, and ferroptosis (115). Because iron is a rate-limiting component for mitochondrial electron transport, manipulating CISD1 expression affects mitochondrial respiratory capacity, beta-oxidation, and oxidative stress (116). CISD1 has been proposed to be a potential chemotherapeutic target as a designed cluvenone derivative (MAD-28) binds to CISD1 and destabilizes its 2Fe-2S cluster. Furthermore, the biological activity of MAD-28 depends on the level of CISD1 in cancer cells. MAD-28 was shown to have high specificity in the selective killing of malignant epithelial breast cancer cells without any apparent effects on normal cells (117).

Cancer cells display uninhibited DNA replication; therefore, DNA polymerases and DNA repair proteins have been exploited as therapeutic targets to combat certain types of cancer (118, 119). Nucleoside reverse transcriptase inhibitor (NRTI)-sensitive mitochondrial DNA polymerases afford a unique opportunity to target cancer cell mitochondria as certain cancers have an increased reliance on OXPHOS, and nDNA polymerases are less sensitive to NRTI inhibition (120, 121). A study comparing normal hematopoietic cells to a panel of 542 primary acute myeloid leukemia (AML) samples discovered that 55% of the AML samples had increased mtDNA biosynthesis gene expression. Upregulated genes included *POLG*, *POLG2*, *POLRMT*, *Twinkle*, *TFAM*, *SSBP1*, *DGUOK*, *TK2*, nucleotide transporters (*SLC25A33*, *SLC25A36*, and *SLC29A3*) and nucleoside kinases (*CMPK1* and *NME1-NME2*). When treated with the NRTI 2’,3’-dideoxycytidine (ddC) AML cells preferentially activated the NRTI and blocked mtDNA replication and OXPHOS compared to hematopoietic cells. Cytotoxicity was preferentially activated in NRTI-treated AML cells, and an AML animal model treated with low doses of ddC (35 and 75 mg/kg/day over 11 days) resulted in decreased mtDNA, decreased mtDNA-encoded cytochrome oxidase subunit 2 (COX2), and induced tumor regression without apparent toxicity (121, 122). As mammalian mtDNA replication occurs independently from the cell cycle (123–127) mtDNA maintenance-disrupting drugs, such as ddC, impair mitochondrial functions in proliferating and non-proliferating cells. We showed that ddC caused mitochondrial dysfunction in hepatocarcinoma-derived proliferating and differentiated HepaRG human cell cultures (128).

Targeting mtDNA maintenance has also been exploited to treat cancer cell lines with mitochondrial-targeted cisplatin. Nucleotide excision repair (NER) machinery repairs cisplatin-nDNA adducts; however, mitochondria lack NER machinery to deal with this damage. Most cancer cells have an increased mitochondrial membrane potential relative to non-cancer cells, and TPP cations are targeted to mitochondria due to their size, lipophilic properties, and delocalized positive charge. An engineered TPP-tagged cisplatin, Platin-M, caused increased cytotoxicity relative to cisplatin-only treatment in several cancer cell models: cisplatin-resistant A2780/CP70 ovarian cancer, prostate cancer PC3 (inherently resistant to cisplatin therapy), and SH-SY5Y neuroblastoma cells. Furthermore, encapsulating Platin-M into specialized nanoparticles enhanced cytotoxicity. SH-SY5Y cells treated with Platin-M and Platin-M encapsulated in nanoparticles were annexin V-positive and propidium iodide-negative, indicative of early apoptosis. Treatment with both Platin-M and Platin-M encapsulated in nanoparticles weakened mitochondrial citrate synthase activity and diminished bioenergetic parameters: spare respiratory capacity, coupling efficiency, and basal respiration. PC3 cells treated separately with cisplatin, Platin-M, and Platin-M encapsulated inside of nanoparticles were subjected to subcellular fractionation, and then platinum concentrations in various fractions were quantified. Cells treated with Platin-M and Platin-M encapsulated in nanoparticles contained platinum-mtDNA adducts, while cells treated with cisplatin contained mostly platinum-nDNA adducts. These findings support that cisplatin is likely released from Platin-M within mitochondria, then binds to mtDNA and inhibits replication (129).

Because somatic mtDNA nonsynonymous mutations associated with different cancers are common and alter their encoded wild-type proteins, these peptides could be immunogenic neo-non-self-epitopes and targetable antigens for cancer immunotherapy. A study utilized a cellular tumor vaccine generated using BALB/c mouse bone marrow-derived dendritic cells (bmDCs) pulsed with different mitochondrial extracts. BALB/c mice were separately immunized with the bmDCs separately treated with 1. mitochondrial extract from mouse kidney renal cortical adenocarcinoma epithelial (RENCA) cells grown in tissue culture, 2. mitochondrial extract derived from RENCA tumors extracted from mice, and 3. mitochondrial extract from kidneys of healthy mice. The RENCA cells were found to harbor *COX1* and *ND5* mtDNA mutations. Mice immunized with the kidney mitochondrial extract did not elicit a protective response. In contrast, mice immunized with the tumor-derived and tissue culture-derived mitochondrial extracts did elicit a protective immune response with 80 and 70% tumor rejection, respectively. A vaccine generated using the mutant COX1 peptide had therapeutic properties like the RENCA mitochondrial extract (130). Other mitochondrial proteins, such as the E2 component of the pyruvate dehydrogenase complex, the MLRQ subunit of complex I, and aconitase, are highly immunogenic. Still, the mechanism conferring increased immunogenicity needs to be better understood. Whether mtDNA mutations have a role in the specific recognition of cancer cells by the immune system is an exciting area of ongoing research (131).

## Can mtDNA serve as an EC biomarker?

Biomarkers (e.g., a tumor-specific molecular characteristic) are biomolecules in tissue or bodily fluids indicating disease. Blood-based biomarkers such as circulating tumor cells, cell-free DNA, proteins, immune cells, and inflammatory parameters are being studied (8). Biomarkers can be predictive, prognostic, diagnostic, or all of the above. Circulating cell-free mtDNA isolated from blood plasma or serum is considered a diagnostic biomarker that can be utilized to determine the presence of breast, ovarian, and testicular cancers (132–134). Prognostic biomarkers are biological features that can be used to predict the course of a disease irrespective of any treatment, e.g., disease recurrence (8, 135). In a prostate cancer study, high levels of a 79 bp fragment of the mtDNA 16 S rRNA gene in serum correlated with prostate-specific antigen recurrence/progression, suggesting circulating mtDNA levels can be used as an independent prognostic prostate cancer biomarker (136). An endometrial endometrioid adenocarcinoma prognostic biomarker is urgently needed as extra-vaginal recurrence is typically incurable (137). Finally, predictive biomarkers can help identify the treatment from which a patient is likely to benefit (138).

As mentioned above, mtDNA is present in the liquid fraction of blood, and plasma cell-free mtDNA is elevated in certain cancers. This plasma mtDNA is likely present in either cell-free mitochondria or encapsulated within extracellular lipid-based systems such as exosomes, microvesicles, platelets, and apoptotic bodies (24). In one study, a group of researchers observed an increase in serum-derived extracellular vesicles (EVs) in pancreatic ductal adenocarcinoma (PDAC) patients compared to non-cancer patients. They further detected more mutations in mtDNA isolated from EVs of PDAC patients compared to non-cancer patients. This finding suggests that serum-derived cell-free mtDNA mutations can be a diagnostic PDAC biomarker (139).

Pathogen DNA and cytosolic mtDNA and nDNA are known to stimulate the innate immune system. The cytosolic cyclic GMP–AMP synthase (cGAS) senses double-stranded DNA fragments and initiates an immune response by activating the stimulator of interferon genes (STING) adaptor protein. The innate immune response involves the production of type I interferons and cytokines, and mtDNA is released via VDAC containing macropores formed on the mitochondrial outer membrane. These macropores are formed in response to tissue damage and mitochondrial stress (140). In a study investigating the pathological significance of EC *POLE* mutations, the exon 9 P286R variant was shown to impede endometrial tumorigenesis by inducing DNA breaks and activating the cGAS-STING signaling pathway. Compared to WT EC cells in a co-culture trans-well migration assay with T cells, co-cultured *POLE* P286R EC cells attracted more migrating T cells, suggesting the mutation could promote anti-tumor immunity (141). In another study using human endometrial stromal cells (HESCs) stimulated with lipopolysaccharide (LPS), cytoplasmic dsDNA, the cGAS-STING pathway, and *IFN-β1*, *IL-1β*, *IL-6*, and *IL-8* gene expression were increased after LPS stimulation. Furthermore, when mtDNA was isolated and extracted from HESCs and then transfected into HESCs, the levels of cGAS-STING pathway proteins increased (142). Similarly, in a study investigating the role of the pro-inflammatory cytokine interleukin-6 (IL-6) on human endometrial adenocarcinoma MFE-296 cells, IL-6 increased the generation of ROS by enhancing NADH oxidase levels and inducing the cellular release of mtDNA. The cellular leakage of mtDNA caused the activation of cGAS-STING signaling and increased the production of extracellular vesicles containing mtDNA (143). Other mechanisms of extruding mtDNA from cells are under active research and could be linked to the cell-to-cell transfer of whole mitochondria. During a mitochondrial transfer process, cells may receive healthy mitochondria to boost metabolic and bioenergetic functions. Alternatively, recipient cells may receive and degrade dysfunctional organelles to purge sick mitochondria from donor cells.

Currently, no evidence-based screening options exist for EC in high-risk individuals or the general population. Diagnosis is generally carried out following investigation of cardinal symptoms of the disease, i.e., post-menopausal bleeding. Endometrial biopsy, transvaginal ultrasound, and hysteroscopy are standard clinical procedures to investigate the disease. Thus, a reliable blood-based biomarker could help provide management strategies to ensure personalized care to the patients at the most significant risk (8). Furthermore, an EC biomarker could be helpful for early detection and to reveal disease recurrence.

We showed that three EC patient tumors harbor tumor-specific somatic mtDNA heteroplasmy (57), and by expanding the number of EC samples in this review to a total of 111, we see that 79% of the samples contained 10 to 90% mtDNA heteroplasmy. Therefore, if cell-free EC tumor-specific mtDNA heteroplasmy is detectable in patients’ blood plasma, we propose that mtDNA mutations could serve as a useful diagnostic biomarker for this devastating disease. A liquid biopsy for EC mtDNA heteroplasmy in the blood would be a non-invasive option to surgical endometrial biopsy, which could provide tumor information in a simple blood draw. Future work will determine whether cell-free EC mtDNA heteroplasmy can be a prognostic or predictive biomarker.

Another potential tool being studied for EC detection is circulating tumor nDNA (ctDNA). In a study of 48 patients (45 with detectable tumor-associated mutations), NGS was performed using 30 hot spot amplicons generated from plasma cell-free DNA extracts, tumor DNA, and white blood cell DNA/buffy coat. Tumor-associated mutations in a panel of only four genes (*CTNNBI*, *KRAS*, *PTEN*, *PIK3CA*) were detected in the plasma DNA extracts of 15 out of 45 patients but not in the matched negative control germline samples, buffy coat (137).

## Conclusions

Data from previous studies show that EC samples harbor tumor-specific heteroplasmic mtDNA mutations. In the single nucleotide variant analysis done here, 30.4% of the mutations occurring in coding genes and tRNA genes are predicted to be inducing/adapting mutations, and nearly a third of the tumor samples contained these mutations between 10 and 90% heteroplasmy. While mutations in the rRNA genes and other non-coding regions are of unknown functional significance, these variants could also be inducing/adapting mutations. The NGS, clinical findings, and in silico predictions of a mutation’s pathogenicity that support a role for a functional mtDNA mutation need to be complimented with experimental models to understand the mechanisms leading to altered mitochondrial activity in cancer (144). Further, the ability to engineer the mtDNA genome (145–148), or deplete cellular mtDNA *in vitro* (149) and use cytoplasmic hybrids (cybrids) to transport mtDNA between cells (37, 150), and the ability to exploit energetic phenotyping technology like the Seahorse Extracellular Flux Analyzer (151, 152), will allow us to determine the impact of inducing/adapting mutations (and recurrent mutations with unknown functional significance) on cellular functions. Additionally, developing cell line models harboring cancer-specific mtDNA mutations will allow *in vitro* testing of the effectiveness of chemotherapeutics, immunotherapeutics, or both.

Mounting evidence supports that mtDNA mutations contribute to carcinogenesis-inducing and environment-adapting metabolic changes that favor cancer cell growth and maintenance. Additionally, other studies show promise for mitochondrial-targeted drugs and immunotherapies. Recent evidence shows cell-free mtDNA is detectable in the blood of cancer patients. An outstanding question in the mitochondrial research field is how mtDNA molecules in lipid-based systems are released into the blood and whether the release mechanism involves cell-to-cell mitochondrial transfer. Also, another question is whether somatic EC mtDNA mutations provide a complementary or better biomarker than nDNA mutations. Regardless of whether the circular polyploid mtDNA genomes are naked or encapsulated within a mitochondrion (or other membrane vesicles), the high-copy number and topological structure of the maternal genome make it an attractive exonuclease-resistant biomolecule for biomarker use. Based on the evidence in the cancer literature presented here, detecting cell-free EC tumor-specific mtDNA heteroplasmy in liquid biopsies may be possible. If mtDNA can be used as a blood-based biomarker, it will help in the early detection of EC and possibly even EC reoccurrence, which is desperately needed for women’s health maintenance.

## Author contributions

PK: Data curation, Visualization, Writing – review & editing. CY: Writing – review & editing. RS: Writing – review & editing. LB: Writing – review & editing. MY: Writing – review & editing, Conceptualization, Data curation, Formal Analysis, Funding acquisition, Investigation, Methodology, Project administration, Resources, Supervision, Visualization, Writing – original draft.

## Supplementary material

Supplementary Table 1 Two hundred and seventy-nine endometrial carcinoma tumor-specific mtDNA variants from 111 samples.

Supplementary Table 2 Twenty-eight endometrial carcinoma tumor-specific mtDNA indels from 25 samples.

## References

[1] Braun MM Overbeek-Wager EA Grumbo RJ. Diagnosis and management of endometrial cancer. Am Fam Physician. (2016) 93:468–74. 26977831

[2] Giaquinto AN Broaddus RR Jemal A Siegel RL. The changing landscape of gynecologic cancer mortality in the United States. Obstet Gynecol. (2022) 139:440–2. doi: 10.1097/AOG.0000000000004676 PMC8857029 35115431

[3] Mirza MR Chase DM Slomovitz BM dePont Christensen R Novak Z Black D. Dostarlimab for primary advanced or recurrent endometrial cancer. N Engl J Med. (2023) 388:2145–58. doi: 10.1056/NEJMoa2216334 36972026

[4] Zhang S Gong TT Liu FH Jiang YT Sun H Ma XX. Global, regional, and national burden of endometrial cancer, 1990-2017: Results from the global burden of disease study, 2017. Front Oncol. (2019) 9:1440. doi: 10.3389/fonc.2019.01440 31921687 PMC6930915

[5] The Surveillance Epidemiology, and End Results (SEER) Program, National Cancer Institute (NCI). Cancer Stat Facts: Uterine Cancer. Bethesda, MD: National Cancer Institute (2024).

[6] Sternberg AK Buck VU Classen-Linke I Leube RE. How mechanical forces change the human endometrium during the menstrual cycle in preparation for embryo implantation. Cells. (2021) 10:1–19. doi: 10.3390/cells10082008 PMC8391722 34440776

[7] Soslow RA Tornos C Park KJ Malpica A Matias-Guiu X Oliva E. Endometrial carcinoma diagnosis: use of FIGO grading and genomic subcategories in clinical practice: recommendations of the international society of gynecological pathologists. Int J Gynecol Pathol. (2019) 38 Suppl 1:S64–74. doi: 10.1097/PGP.0000000000000518 PMC6295928 30550484

[8] Njoku K Barr CE Crosbie EJ. Current and emerging prognostic biomarkers in endometrial cancer. Front Oncol. (2022) 12:890908. doi: 10.3389/fonc.2022.890908 35530346 PMC9072738

[9] Musicco C Cormio G Pesce V Loizzi V Cicinelli E Resta L. Mitochondrial dysfunctions in type I endometrial carcinoma: Exploring their role in oncogenesis and tumor progression. Int J Mol Sci. (2018) 19:1–14. doi: 10.3390/ijms19072076 PMC6073675 30018222

[10] Stewart BW Wild C. International agency for research on cancer, world health organization. In: World cancer report 2014. WHO Press, Lyon, France; Geneva, Switzerland (2014). 630.

[11] Leslie KK Thiel KW Goodheart MJ De Geest K Jia Y Yang S. Endometrial cancer. Obstet Gynecol Clin North Am. (2012) 39:255–68. doi: 10.1016/j.ogc.2012.04.001 PMC3518445 22640714

[12] Setiawan VW Yang HP Pike MC McCann SE Yu H Xiang YB. Type I and II endometrial cancers: have they different risk factors? J Clin Oncol. (2013) 31:2607–18. doi: 10.1200/JCO.2012.48.2596 PMC3699726 23733771

[13] Zahnd WE Hyon KS Diaz-Sylvester P Izadi SR Colditz GA Brard L. Rural-urban differences in surgical treatment, regional lymph node examination, and survival in endometrial cancer patients. Cancer Causes Control. (2018) 29:221–32. doi: 10.1007/s10552-017-0998-4 PMC6311991 29282582

[14] Cancer Genome Atlas Research N Kandoth C Schultz N Cherniack AD Akbani R Liu Y. Integrated genomic characterization of endometrial carcinoma. Nature. (2013) 497:67–73. doi: 10.1038/nature12113 23636398 PMC3704730

[15] Berek JS Matias-Guiu X Creutzberg C Fotopoulou C Gaffney D Kehoe S. FIGO staging of endometrial cancer: 2023. Int J Gynaecol Obstet. (2023) 162:383–94. doi: 10.1002/ijgo.14923 37337978

[16] Berek JS Matias-Guiu X Creutzberg C Fotopoulou C Gaffney D Kehoe S. Correction to "FIGO staging of endometrial cancer". Int J Gynaecol Obstet. (2023). doi: 10.1002/ijgo.15193 38055215

[17] Yuan Y Ju YS Kim Y Li J Wang Y Yoon CJ. Comprehensive molecular characterization of mitochondrial genomes in human cancers. Nat Genet. (2020) 52:342–52. doi: 10.1038/s41588-019-0557-x PMC7058535 32024997

[18] Selvaraj V Stocco DM Clark BJ. Current knowledge on the acute regulation of steroidogenesis. Biol Reprod. (2018) 99:13–26. doi: 10.1093/biolre/ioy102 29718098 PMC6044331

[19] Young MJ Copeland WC. Human mitochondrial DNA replication machinery and disease. Curr Opin Genet Dev. (2016) 38:52–62. doi: 10.1016/j.gde.2016.03.005 27065468 PMC5055853

[20] Minasyan L Sreekumar PG Hinton DR Kannan R. Protective mechanisms of the mitochondrial-derived peptide humanin in oxidative and endoplasmic reticulum stress in RPE cells. Oxid Med Cell Longev. (2017) 2017:1675230. doi: 10.1155/2017/1675230 28814984 PMC5549471

[21] Kim SJ Xiao J Wan J Cohen P Yen K. Mitochondrially derived peptides as novel regulators of metabolism. J Physiol. (2017) 595:6613–21. doi: 10.1113/JP274472 PMC5663826 28574175

[22] Moya GE Rivera PD Dittenhafer-Reed KE. Evidence for the role of mitochondrial DNA release in the inflammatory response in neurological disorders. Int J Mol Sci. (2021) 22:1–26. doi: 10.3390/ijms22137030 PMC8268735 34209978

[23] Lee C Zeng J Drew BG Sallam T Martin-Montalvo A Wan J. The mitochondrial-derived peptide MOTS-c promotes metabolic homeostasis and reduces obesity and insulin resistance. Cell Metab. (2015) 21:443–54. doi: 10.1016/j.cmet.2015.02.009 PMC4350682 25738459

[24] Trumpff C Michelson J Lagranha CJ Taleon V Karan KR Sturm G. Stress and circulating cell-free mitochondrial DNA: A systematic review of human studies, physiological considerations, and technical recommendations. Mitochondrion. (2021) 59:225–45. doi: 10.1016/j.mito.2021.04.002 PMC8418815 33839318

[25] Warburg O. On the origin of cancer cells. Science. (1956) 123:309–14. doi: 10.1126/science.123.3191.309 13298683

[26] Liberti MV Locasale JW. The warburg effect: How does it benefit cancer cells? Trends Biochem Sci. (2016) 41:211–8. doi: 10.1016/j.tibs.2015.12.001 PMC4783224 26778478

[27] Weinberg SE Chandel NS. Targeting mitochondria metabolism for cancer therapy. Nat Chem Biol. (2015) 11:9–15. doi: 10.1038/nchembio.1712 25517383 PMC4340667

[28] Battogtokh G Choi YS Kang DS Park SJ Shim MS Huh KM. Mitochondria-targeting drug conjugates for cytotoxic, anti-oxidizing and sensing purposes: current strategies and future perspectives. Acta Pharm Sin B. (2018) 8:862–80. doi: 10.1016/j.apsb.2018.05.006 PMC6251809 30505656

[29] Pavlova NN Zhu J Thompson CB. The hallmarks of cancer metabolism: Still emerging. Cell Metab. (2022) 34:355–77. doi: 10.1016/j.cmet.2022.01.007 PMC8891094 35123658

[30] Lau AN Li Z Danai LV Westermark AM Darnell AM Ferreira R. Dissecting cell-type-specific metabolism in pancreatic ductal adenocarcinoma. Elife. (2020) 9:1–35. doi: 10.7554/eLife.56782 PMC7406355 32648540

[31] Mullen AR Wheaton WW Jin ES Chen PH Sullivan LB Cheng T. Reductive carboxylation supports growth in tumour cells with defective mitochondria. Nature. (2011) 481:385–8. doi: 10.1038/nature10642 PMC3262117 22101431

[32] Mahmood M Liu EM Shergold AL Tolla E Tait-Mulder J Huerta-Uribe A. Mitochondrial DNA mutations drive aerobic glycolysis to enhance checkpoint blockade response in melanoma. Nat Cancer. (2024) 5:659–672. doi: 10.1038/s43018-023-00721-w PMC11056318 38286828

[33] Titov DV Cracan V Goodman RP Peng J Grabarek Z Mootha VK. Complementation of mitochondrial electron transport chain by manipulation of the NAD+/NADH ratio. Science. (2016) 352:231–5. doi: 10.1126/science.aad4017 PMC4850741 27124460

[34] Evans DR Guy HI. Mammalian pyrimidine biosynthesis: fresh insights into an ancient pathway. J Biol Chem. (2004) 279:33035–8. doi: 10.1074/jbc.R400007200 15096496

[35] Dickinson ME Flenniken AM Ji X Teboul L Wong MD White JK. High-throughput discovery of novel developmental phenotypes. Nature. (2016) 537:508–14. doi: 10.1038/nature19356 PMC5295821 27626380

[36] Khutornenko AA Roudko VV Chernyak BV Vartapetian AB Chumakov PM Evstafieva AG. Pyrimidine biosynthesis links mitochondrial respiration to the p53 pathway. Proc Natl Acad Sci U S A. (2010) 107:12828–33. doi: 10.1073/pnas.0910885107 PMC2919937 20566882

[37] Schon EA DiMauro S Hirano M. Human mitochondrial DNA: roles of inherited and somatic mutations. Nat Rev Genet. (2012) 13:878–90. doi: 10.1038/nrg3275 PMC3959762 23154810

[38] Weinberg F Hamanaka R Wheaton WW Weinberg S Joseph J Lopez M. Mitochondrial metabolism and ROS generation are essential for Kras-mediated tumorigenicity. Proc Natl Acad Sci U S A. (2010) 107:8788–93. doi: 10.1073/pnas.1003428107 PMC2889315 20421486

[39] Guerra F Kurelac I Cormio A Zuntini R Amato LB Ceccarelli C. Placing mitochondrial DNA mutations within the progression model of type I endometrial carcinoma. Hum Mol Genet. (2011) 20:2394–405. doi: 10.1093/hmg/ddr146 21470976

[40] Young MJ Humble MM DeBalsi KL Sun KY Copeland WC. POLG2 disease variants: analyses reveal a dominant negative heterodimer, altered mitochondrial localization and impaired respiratory capacity. Hum Mol Genet. (2015) 24:5184–97. doi: 10.1093/hmg/ddv240 PMC4550827 26123486

[41] Jayaprakash AD Benson EK Gone S Liang R Shim J Lambertini L. Stable heteroplasmy at the single-cell level is facilitated by intercellular exchange of mtDNA. Nucleic Acids Res. (2015) 43:2177–87. doi: 10.1093/nar/gkv052 PMC4344500 25653158

[42] He Y Wu J Dressman DC Iacobuzio-Donahue C Markowitz SD Velculescu VE. Heteroplasmic mitochondrial DNA mutations in normal and tumour cells. Nature. (2010) 464:610–4. doi: 10.1038/nature08802 PMC3176451 20200521

[43] Kopinski PK Singh LN Zhang S Lott MT Wallace DC. Mitochondrial DNA variation and cancer. Nat Rev Cancer. (2021) 21:431–45. doi: 10.1038/s41568-021-00358-w 34045735

[44] Gammage PA Frezza C. Mitochondrial DNA: the overlooked oncogenome? BMC Biol. (2019) 17:53. doi: 10.1186/s12915-019-0668-y 31286943 PMC6615100

[45] Spinelli JB Haigis MC. The multifaceted contributions of mitochondria to cellular metabolism. Nat Cell Biol. (2018) 20:745–54. doi: 10.1038/s41556-018-0124-1 PMC6541229 29950572

[46] Fujii J Homma T Kobayashi S Warang P Madkaikar M Mukherjee MB. Erythrocytes as a preferential target of oxidative stress in blood. Free Radic Res. (2021) 55:562–80. doi: 10.1080/10715762.2021.1873318 33427524

[47] Hance N Ekstrand MI Trifunovic A. Mitochondrial DNA polymerase gamma is essential for mammalian embryogenesis. Hum Mol Genet. (2005) 14:1775–83. doi: 10.1093/hmg/ddi184 15888483

[48] Iyengar B Luo N Farr CL Kaguni LS Campos AR. The accessory subunit of DNA polymerase gamma is essential for mitochondrial DNA maintenance and development in Drosophila melanogaster. Proc Natl Acad Sci U S A. (2002) 99:4483–8. doi: 10.1073/pnas.072664899 PMC123674 11917141

[49] Humble MM Young MJ Foley JF Pandiri AR Travlos GS Copeland WC. Polg2 is essential for mammalian embryogenesis and is required for mtDNA maintenance. Hum Mol Genet. (2013) 22:1017–25. doi: 10.1093/hmg/dds506 PMC3561914 23197651

[50] Lee SK Zhao MH Zheng Z Kwon JW Liang S Kim SH. Polymerase subunit gamma 2 affects porcine oocyte maturation and subsequent embryonic development. Theriogenology. (2014) 83:121–30. doi: 10.1016/j.theriogenology.2014.08.019 25308052

[51] Cerritelli SM Frolova EG Feng C Grinberg A Love PE Crouch RJ. Failure to produce mitochondrial DNA results in embryonic lethality in Rnaseh1 null mice. Mol Cell. (2003) 11:807–15. doi: 10.1016/S1097-2765(03)00088-1 12667461

[52] Tyynismaa H Mjosund KP Wanrooij S Lappalainen I Ylikallio E Jalanko A. Mutant mitochondrial helicase Twinkle causes multiple mtDNA deletions and a late-onset mitochondrial disease in mice. Proc Natl Acad Sci U S A. (2005) 102:17687–92. doi: 10.1073/pnas.0505551102 PMC1308896 16301523

[53] Tyynismaa H Suomalainen A. Mouse models of mitochondrial DNA defects and their relevance for human disease. EMBO Rep. (2009) 10:137–43. doi: 10.1038/embor.2008.242 PMC2637315 19148224

[54] Rahman MM Young CKJ Goffart S Pohjoismaki JLO Young MJ Heterozygous P. Y955C mutation in DNA polymerase gamma leads to alterations in bioenergetics, complex I subunit expression, and mtDNA replication. J Biol Chem. (2022) 298:102196. doi: 10.1016/j.jbc.2022.102196 35760101 PMC9307957

[55] Bajzikova M Kovarova J Coelho AR Boukalova S Oh S Rohlenova K. Reactivation of dihydroorotate dehydrogenase-driven pyrimidine biosynthesis restores tumor growth of respiration-deficient cancer cells. Cell Metab. (2019) 29:399–416.e10. doi: 10.1016/j.cmet.2018.10.014 30449682 PMC7484595

[56] Gorelick AN Kim M Chatila WK La K Hakimi AA Berger MF. Respiratory complex and tissue lineage drive recurrent mutations in tumour mtDNA. Nat Metab. (2021) 3:558–70. doi: 10.1038/s42255-021-00378-8 PMC9304985 33833465

[57] Young MJ Sachidanandam R Hales DB Brard L Robinson K Rahman MM. Identification of somatic mitochondrial DNA mutations, heteroplasmy, and increased levels of catenanes in tumor specimens obtained from three endometrial cancer patients. Life (Basel). (2022) 12:1–20. doi: 10.3390/life12040562 PMC9030153 35455053

[58] Mattiazzi M Vijayvergiya C Gajewski CD DeVivo DC Lenaz G Wiedmann M. The mtDNA T8993G (NARP) mutation results in an impairment of oxidative phosphorylation that can be improved by antioxidants. Hum Mol Genet. (2004) 13:869–79. doi: 10.1093/hmg/ddh103 14998933

[59] Taylor RW Turnbull DM. Mitochondrial DNA mutations in human disease. Nat Rev Genet. (2005) 6:389–402. doi: 10.1038/nrg1606 15861210 PMC1762815

[60] Wallace DC Chalkia D. Mitochondrial DNA genetics and the heteroplasmy conundrum in evolution and disease. Cold Spring Harb Perspect Biol. (2013) 5:a021220. doi: 10.1101/cshperspect.a021220 24186072 PMC3809581

[61] Ju YS Alexandrov LB Gerstung M Martincorena I Nik-Zainal S Ramakrishna M. Origins and functional consequences of somatic mitochondrial DNA mutations in human cancer. Elife. (2014) 3:1–28. doi: 10.7554/eLife.02935 PMC4371858 25271376

[62] Wallace DC Fan W Procaccio V. Mitochondrial energetics and therapeutics. Annu Rev Pathol. (2010) 5:297–348. doi: 10.1146/annurev.pathol.4.110807.092314 20078222 PMC3245719

[63] Zong WX Rabinowitz JD White E. Mitochondria and cancer. Mol Cell. (2016) 61:667–76. doi: 10.1016/j.molcel.2016.02.011 PMC4779192 26942671

[64] Grandhi S Bosworth C Maddox W Sensiba C Akhavanfard S Ni Y. Heteroplasmic shifts in tumor mitochondrial genomes reveal tissue-specific signals of relaxed and positive selection. Hum Mol Genet. (2017) 26:2912–22. doi: 10.1093/hmg/ddx172 PMC5886292 28475717

[65] Falkenberg M Gustafsson CM. Mammalian mitochondrial DNA replication and mechanisms of deletion formation. Crit Rev Biochem Mol Biol. (2020) 55:509–24. doi: 10.1080/10409238.2020.1818684 32972254

[66] Gustafsson CM Falkenberg M Larsson NG. Maintenance and expression of mammalian mitochondrial DNA. Annu Rev Biochem. (2016) 85:133–60. doi: 10.1146/annurev-biochem-060815-014402 27023847

[67] Shadel GS Clayton DA. Mitochondrial DNA maintenance in vertebrates. Annu Rev Biochem. (1997) 66:409–35. doi: 10.1146/annurev.biochem.66.1.409 9242913

[68] Clayton DA. Replication of animal mitochondrial DNA. Cell. (1982) 28:693–705. doi: 10.1016/0092-8674(82)90049-6 6178513

[69] Taanman JW. The mitochondrial genome: structure, transcription, translation and replication. Biochim Biophys Acta. (1999) 1410:103–23. doi: 10.1016/S0005-2728(98)00161-3 10076021

[70] Lawless C Greaves L Reeve AK Turnbull DM Vincent AE. The rise and rise of mitochondrial DNA mutations. Open Biol. (2020) 10:200061. doi: 10.1098/rsob.200061 32428418 PMC7276526

[71] Belle EM Piganeau G Gardner M Eyre-Walker A. An investigation of the variation in the transition bias among various animal mitochondrial DNA. Gene. (2005) 355:58–66. doi: 10.1016/j.gene.2005.05.019 16039074

[72] Lott MT Leipzig JN Derbeneva O Xie HM Chalkia D Sarmady M. mtDNA variation and analysis using mitomap and mitomaster. Curr Protoc Bioinf. (2013) 44:1 23 1–6. doi: 10.1002/0471250953.bi0123s44 PMC4257604 25489354

[73] Gudmundsson S Singer-Berk M Watts NA Phu W Goodrich JK Solomonson M. Variant interpretation using population databases: Lessons from gnomAD. Hum Mutat. (2022) 43:1012–30. doi: 10.1002/humu.24309 PMC9160216 34859531

[74] Bolze A Mendez F White S Tanudjaja F Isaksson M Rashkin M. Selective constraints and pathogenicity of mitochondrial DNA variants inferred from a novel database of 196,554 unrelated individuals. bioRxiv. (2019). doi: 10.1101/798264

[75] Sonney S Leipzig J Lott MT Zhang S Procaccio V Wallace DC. Predicting the pathogenicity of novel variants in mitochondrial tRNA with MitoTIP. PLoS Comput Biol. (2017) 13:e1005867. doi: 10.1371/journal.pcbi.1005867 29227991 PMC5739504

[76] Corona P Lamantea E Greco M Carrara F Agostino A Guidetti D. Novel heteroplasmic mtDNA mutation in a family with heterogeneous clinical presentations. Ann Neurol. (2002) 51:118–22. doi: 10.1002/ana.10059 11782991

[77] Lim AZ Blakely EL Baty K He L Hopton S Falkous G. A novel pathogenic m.4412G>A MT-TM mitochondrial DNA variant associated with childhood-onset seizures, myopathy and bilateral basal ganglia changes. Mitochondrion. (2019) 47:18–23. doi: 10.1016/j.mito.2019.04.007 31022467 PMC6617384

[78] Castellana S Biagini T Petrizzelli F Parca L Panzironi N Caputo V. MitImpact 3: modeling the residue interaction network of the Respiratory Chain subunits. Nucleic Acids Res. (2021) 49:D1282–D8. doi: 10.1093/nar/gkaa1032 PMC7779045 33300029

[79] Castellana S Fusilli C Mazzoccoli G Biagini T Capocefalo D Carella M. High-confidence assessment of functional impact of human mitochondrial non-synonymous genome variations by APOGEE. PLoS Comput Biol. (2017) 13:e1005628. doi: 10.1371/journal.pcbi.1005628 28640805 PMC5501658

[80] Bianco SD Parca L Petrizzelli F Biagini T Giovannetti A Liorni N. APOGEE 2: multi-layer machine-learning model for the interpretable prediction of mitochondrial missense variants. Nat Commun. (2023) 14:5058. doi: 10.1038/s41467-023-40797-7 37598215 PMC10439926

[81] Reva B Antipin Y Sander C. Predicting the functional impact of protein mutations: application to cancer genomics. Nucleic Acids Res. (2011) 39:e118. doi: 10.1093/nar/gkr407 21727090 PMC3177186

[82] Schmitt MW Kennedy SR Salk JJ Fox EJ Hiatt JB Loeb LA. Detection of ultra-rare mutations by next-generation sequencing. Proc Natl Acad Sci U S A. (2012) 109:14508–13. doi: 10.1073/pnas.1208715109 PMC3437896 22853953

[83] Song S Pursell ZF Copeland WC Longley MJ Kunkel TA Mathews CK. DNA percursor asymmetries in mammalian tissue mitochondrial and possible contribution to mitochondrial mutagenesis through reduced replication fidleity. Proc Natl Acad Sci U S A. (2005) 102:4990–5. doi: 10.1073/pnas.0500253102 PMC555996 15784738

[84] Lindahl T. Instability and decay of the primary structure of DNA. Nature. (1993) 362:709–15. doi: 10.1038/362709a0 8469282

[85] Bohr VA. Repair of oxidative DNA damage in nuclear and mitochondrial DNA, and some changes with aging in mammalian cells. Free Radic Biol Med. (2002) 32:804–12. doi: 10.1016/S0891-5849(02)00787-6 11978482

[86] Jaberi E Tresse E Gronbaek K Weischenfeldt J Issazadeh-Navikas S. Identification of unique and shared mitochondrial DNA mutations in neurodegeneration and cancer by single-cell mitochondrial DNA structural variation sequencing (MitoSV-seq). EBioMedicine. (2020) 57:102868. doi: 10.1016/j.ebiom.2020.102868 32629384 PMC7334819

[87] Clay Montier LL Deng JJ Bai Y. Number matters: control of mammalian mitochondrial DNA copy number. J Genet Genomics. (2009) 36:125–31. doi: 10.1016/S1673-8527(08)60099-5 PMC4706993 19302968

[88] Rosa HS Ajaz S Gnudi L Malik AN. A case for measuring both cellular and cell-free mitochondrial DNA as a disease biomarker in human blood. FASEB J. (2020) 34:12278–88. doi: 10.1096/fj.202000959RR 32729179

[89] Wai T Ao A Zhang X Cyr D Dufort D Shoubridge EA. The role of mitochondrial DNA copy number in mammalian fertility. Biol Reprod. (2010) 83:52–62. doi: 10.1095/biolreprod.109.080887 20130269 PMC2888963

[90] Sun Y Zhang L Ho SS Wu X Gu J. Lower mitochondrial DNA copy number in peripheral blood leukocytes increases the risk of endometrial cancer. Mol Carcinog. (2016) 55:1111–7. doi: 10.1002/mc.22373 26258624

[91] Hosgood HD 3rd Liu CS Rothman N Weinstein SJ Bonner MR Shen M. Mitochondrial DNA copy number and lung cancer risk in a prospective cohort study. Carcinogenesis. (2010) 31:847–9. doi: 10.1093/carcin/bgq045 PMC2864414 20176654

[92] Shen J Platek M Mahasneh A Ambrosone CB Zhao H. Mitochondrial copy number and risk of breast cancer: a pilot study. Mitochondrion. (2010) 10:62–8. doi: 10.1016/j.mito.2009.09.004 PMC5040184 19788937

[93] Xing J Chen M Wood CG Lin J Spitz MR Ma J. Mitochondrial DNA content: its genetic heritability and association with renal cell carcinoma. J Natl Cancer Inst. (2008) 100:1104–12. doi: 10.1093/jnci/djn213 PMC2720693 18664653

[94] Xie H Lev D Gong Y Wang S Pollock RE Wu X. Reduced mitochondrial DNA copy number in peripheral blood leukocytes increases the risk of soft tissue sarcoma. Carcinogenesis. (2013) 34:1039–43. doi: 10.1093/carcin/bgt023 23349016

[95] Xu E Sun W Gu J Chow WH Ajani JA Wu X. Association of mitochondrial DNA copy number in peripheral blood leukocytes with risk of esophageal adenocarcinoma. Carcinogenesis. (2013) 34:2521–4. doi: 10.1093/carcin/bgt230 PMC3810839 23803692

[96] Cormio A Cormio G Musicco C Sardanelli AM Gasparre G Gadaleta MN. Mitochondrial changes in endometrial carcinoma: possible role in tumor diagnosis and prognosis (review). Oncol Rep. (2015) 33:1011–8. doi: 10.3892/or.2014.3690 25530491

[97] Wang Y Liu VW Xue WC Tsang PC Cheung AN Ngan HY. The increase of mitochondrial DNA content in endometrial adenocarcinoma cells: a quantitative study using laser-captured microdissected tissues. Gynecol Oncol. (2005) 98:104–10. doi: 10.1016/j.ygyno.2005.04.015 15921730

[98] Cormio A Guerra F Cormio G Pesce V Fracasso F Loizzi V. Mitochondrial DNA content and mass increase in progression from normal to hyperplastic to cancer endometrium. BMC Res Notes. (2012) 5:279. doi: 10.1186/1756-0500-5-279 22676897 PMC3502111

[99] Xu L Hu Y Chen B Tang W Han X Yu H. Mitochondrial polymorphisms as risk factors for endometrial cancer in southwest China. Int J Gynecol Cancer. (2006) 16:1661–7. doi: 10.1136/ijgc-00009577-200607000-00026 16884381

[100] Czarnecka AM Klemba A Semczuk A Plak K Marzec B Krawczyk T. Common mitochondrial polymorphisms as risk factor for endometrial cancer. Int Arch Med. (2009) 2:33. doi: 10.1186/1755-7682-2-33 19863780 PMC2775024

[101] Brown AJ Westin SN Broaddus RR Schmeler K. Progestin intrauterine device in an adolescent with grade 2 endometrial cancer. Obstet Gynecol. (2012) 119:423–6. doi: 10.1097/AOG.0b013e318234d97c PMC3266511 22270425

[102] Novikova OV Nosov VB Panov VA Novikova EG Krasnopolskaya KV Andreeva YY. Live births and maintenance with levonorgestrel IUD improve disease-free survival after fertility-sparing treatment of atypical hyperplasia and early endometrial cancer. Gynecol Oncol. (2021) 161:152–9. doi: 10.1016/j.ygyno.2021.01.001 33461741

[103] Westin SN Fellman B Sun CC Broaddus RR Woodall ML Pal N. Prospective phase II trial of levonorgestrel intrauterine device: nonsurgical approach for complex atypical hyperplasia and early-stage endometrial cancer. Am J Obstet Gynecol. (2021) 224:191.e1–e15. doi: 10.1016/j.ajog.2020.08.032 PMC7855308 32805208

[104] Altman AD Thompson J Nelson G Chu P Nation J Ghatage P. Use of aromatase inhibitors as first- and second-line medical therapy in patients with endometrial adenocarcinoma: a retrospective study. J Obstet Gynaecol Can. (2012) 34:664–72. doi: 10.1016/s1701-2163(16)35320-8 22742486

[105] Barker LC Brand IR Crawford SM. Sustained effect of the aromatase inhibitors anastrozole and letrozole on endometrial thickness in patients with endometrial hyperplasia and endometrial carcinoma. Curr Med Res Opin. (2009) 25:1105–9. doi: 10.1185/03007990902860549 19301987

[106] Fiorica JV Brunetto VL Hanjani P Lentz SS Mannel R Andersen W. Phase II trial of alternating courses of megestrol acetate and tamoxifen in advanced endometrial carcinoma: a Gynecologic Oncology Group study. Gynecol Oncol. (2004) 92:10–4. doi: 10.1016/j.ygyno.2003.11.008 14751131

[107] Whitney CW Brunetto VL Zaino RJ Lentz SS Sorosky J Armstrong DK. Phase II study of medroxyprogesterone acetate plus tamoxifen in advanced endometrial carcinoma: a Gynecologic Oncology Group study. Gynecol Oncol. (2004) 92:4–9. doi: 10.1016/j.ygyno.2003.09.018 14751130

[108] Singh M Zaino RJ Filiaci VJ Leslie KK. Relationship of estrogen and progesterone receptors to clinical outcome in metastatic endometrial carcinoma: a Gynecologic Oncology Group Study. Gynecol Oncol. (2007) 106:325–33. doi: 10.1016/j.ygyno.2007.03.042 17532033

[109] Kauppila A. Oestrogen and progestin receptors as prognostic indicators in endometrial cancer. A Rev literature. Acta Oncol. (1989) 28:561–6. doi: 10.3109/02841868909092271 2675940

[110] Thigpen JT Brady MF Alvarez RD Adelson MD Homesley HD Manetta A. Oral medroxyprogesterone acetate in the treatment of advanced or recurrent endometrial carcinoma: a dose-response study by the Gynecologic Oncology Group. J Clin Oncol. (1999) 17:1736–44. doi: 10.1200/JCO.1999.17.6.1736 10561210

[111] Dellinger TH Monk BJ. Systemic therapy for recurrent endometrial cancer: a review of North American trials. Expert Rev Anticancer Ther. (2009) 9:905–16. doi: 10.1586/era.09.54 19589030

[112] Slomovitz BM Filiaci VL Walker JL Taub MC Finkelstein KA Moroney JW. A randomized phase II trial of everolimus and letrozole or hormonal therapy in women with advanced, persistent or recurrent endometrial carcinoma: A GOG Foundation study. Gynecol Oncol. (2022) 164:481–91. doi: 10.1016/j.ygyno.2021.12.031 35063278

[113] Eskander RN Sill MW Beffa L Moore RG Hope JM Musa FB. Pembrolizumab plus chemotherapy in advanced endometrial cancer. N Engl J Med. (2023) 388:2159–70. doi: 10.1056/NEJMoa2302312 PMC10351614 36972022

[114] Dhanasekaran S Venugopal D Al-Dayan N Ravinayagam V Mohammed AA. Emerging insights into mitochondria-specific targeting and drug delivering strategies: Recent milestones and therapeutic implications. Saudi J Biol Sci. (2020) 27:3581–92. doi: 10.1016/j.sjbs.2020.07.030 PMC7714987 33304169

[115] Yuan H Li X Zhang X Kang R Tang D. CISD1 inhibits ferroptosis by protection against mitochondrial lipid peroxidation. Biochem Biophys Res Commun. (2016) 478:838–44. doi: 10.1016/j.bbrc.2016.08.034 27510639

[116] Kusminski CM Holland WL Sun K Park J Spurgin SB Lin Y. MitoNEET-driven alterations in adipocyte mitochondrial activity reveal a crucial adaptive process that preserves insulin sensitivity in obesity. Nat Med. (2012) 18:1539–49. doi: 10.1038/nm.2899 PMC3745511 22961109

[117] Bai F Morcos F Sohn YS Darash-Yahana M Rezende CO Lipper CH. The Fe-S cluster-containing NEET proteins mitoNEET and NAF-1 as chemotherapeutic targets in breast cancer. Proc Natl Acad Sci U S A. (2015) 112:3698–703. doi: 10.1073/pnas.1502960112 PMC4378444 25762074

[118] Somasagara RR Spencer SM Tripathi K Clark DW Mani C Madeira da Silva L. RAD6 promotes DNA repair and stem cell signaling in ovarian cancer and is a promising therapeutic target to prevent and treat acquired chemoresistance. Oncogene. (2017) 36:6680–90. doi: 10.1038/onc.2017.279 PMC5709226 28806395

[119] Lange SS Takata K Wood RD. DNA polymerases and cancer. Nat Rev Cancer. (2011) 11:96–110. doi: 10.1038/nrc2998 21258395 PMC3739438

[120] Martin JL Brown CE Matthews-Davis N Reardon JE. Effects of antiviral nucleoside analogs on human DNA polymerases and mitochondrial DNA synthesis. Antimicrob Agents Chemother. (1994) 38:2743–9. doi: 10.1128/AAC.38.12.2743 PMC188279 7695256

[121] Liyanage SU Hurren R Voisin V Bridon G Wang X Xu C. Leveraging increased cytoplasmic nucleoside kinase activity to target mtDNA and oxidative phosphorylation in AML. Blood. (2017) 129:2657–66. doi: 10.1182/blood-2016-10-741207 PMC5766841 28283480

[122] Young MJ. Off-target effects of drugs that disrupt human mitochondrial DNA maintenance. Front Mol Biosci. (2017) 4:74. doi: 10.3389/fmolb.2017.00074 29214156 PMC5702650

[123] Bogenhagen D Clayton DA. Mouse L cell mitochondrial DNA molecules are selected randomly for replication throughout the cell cycle. Cell. (1977) 11:719–27. doi: 10.1016/0092-8674(77)90286-0 560914

[124] Pontarin G Fijolek A Pizzo P Ferraro P Rampazzo C Pozzan T. Ribonucleotide reduction is a cytosolic process in mammalian cells independently of DNA damage. Proc Natl Acad Sci U S A. (2008) 105:17801–6. doi: 10.1073/pnas.0808198105 PMC2584719 18997010

[125] Saada A Shaag A Elpeleg O. mtDNA depletion myopathy: elucidation of the tissue specificity in the mitochondrial thymidine kinase (TK2) deficiency. Mol Genet Metab. (2003) 79:1–5. doi: 10.1016/S1096-7192(03)00063-5 12765840

[126] Papa S. Mitochondrial oxidative phosphorylation changes in the life span. Molecular aspects and physiopathological implications. Biochim Biophys Acta. (1996) 1276:87–105. doi: 10.1016/0005-2728(96)00077-1 8816944

[127] Saada A Shaag A Mandel H Nevo Y Eriksson S Elpeleg O. Mutant mitochondrial thymidine kinase in mitochondrial DNA depletion myopathy. Nat Genet. (2001) 29:342–4. doi: 10.1038/ng751 11687801

[128] Young CKJ Wheeler JH Rahman MM Young MJ. The antiretroviral 2',3'-dideoxycytidine causes mitochondrial dysfunction in proliferating and differentiated HepaRG human cell cultures. J Biol Chem. (2021) 296:100206. doi: 10.1074/jbc.RA120.014885 33334881 PMC7948951

[129] Marrache S Pathak RK Dhar S. Detouring of cisplatin to access mitochondrial genome for overcoming resistance. Proc Natl Acad Sci U S A. (2014) 111:10444–9. doi: 10.1073/pnas.1405244111 PMC4115573 25002500

[130] Pierini S Fang C Rafail S Facciponte JG Huang J De Sanctis F. A tumor mitochondria vaccine protects against experimental renal cell carcinoma. J Immunol. (2015) 195:4020–7. doi: 10.4049/jimmunol.1500281 26378078

[131] Prota G Lechuga-Vieco AV De Libero G. Mitochondrial proteins as source of cancer neoantigens. Int J Mol Sci. (2022) 23:1–10. doi: 10.3390/ijms23052627 PMC8909979 35269772

[132] Kohler C Radpour R Barekati Z Asadollahi R Bitzer J Wight E. Levels of plasma circulating cell free nuclear and mitochondrial DNA as potential biomarkers for breast tumors. Mol Cancer. (2009) 8:105. doi: 10.1186/1476-4598-8-105 19922604 PMC2780981

[133] Zachariah RR Schmid S Buerki N Radpour R Holzgreve W Zhong X. Levels of circulating cell-free nuclear and mitochondrial DNA in benign and Malignant ovarian tumors. Obstet Gynecol. (2008) 112:843–50. doi: 10.1097/AOG.0b013e3181867bc0 18827127

[134] Ellinger J Albers P Muller SC von Ruecker A Bastian PJ. Circulating mitochondrial DNA in the serum of patients with testicular germ cell cancer as a novel noninvasive diagnostic biomarker. BJU Int. (2009) 104:48–52. doi: 10.1111/j.1464-410X.2008.08289.x 19154496

[135] Sechidis K Papangelou K Metcalfe PD Svensson D Weatherall J Brown G. Distinguishing prognostic and predictive biomarkers: an information theoretic approach. Bioinformatics. (2018) 34:3365–76. doi: 10.1093/bioinformatics/bty357 PMC6157098 29726967

[136] Ellinger J Muller SC Wernert N von Ruecker A Bastian PJ. Mitochondrial DNA in serum of patients with prostate cancer: a predictor of biochemical recurrence after prostatectomy. BJU Int. (2008) 102:628–32. doi: 10.1111/j.1464-410X.2008.07613.x 18410441

[137] Bolivar AM Luthra R Mehrotra M Chen W Barkoh BA Hu P. Targeted next-generation sequencing of endometrial cancer and matched circulating tumor DNA: identification of plasma-based, tumor-associated mutations in early stage patients. Mod Pathol. (2019) 32:405–14. doi: 10.1038/s41379-018-0158-8 PMC6395490 30315273

[138] Shaw A Bradley MD Elyan S Kurian KM. Tumour biomarkers: diagnostic, prognostic, and predictive. BMJ. (2015) 351:h3449. doi: 10.1136/bmj.h3449 26141725

[139] Vikramdeo KS Anand S Khan MA Khushman M Heslin MJ Singh S. Detection of mitochondrial DNA mutations in circulating mitochondria-originated extracellular vesicles for potential diagnostic applications in pancreatic adenocarcinoma. Sci Rep. (2022) 12:18455. doi: 10.1038/s41598-022-22006-5 36323735 PMC9630429

[140] Kim J Kim HS Chung JH. Molecular mechanisms of mitochondrial DNA release and activation of the cGAS-STING pathway. Exp Mol Med. (2023) 55:510–9. doi: 10.1038/s12276-023-00965-7 PMC10037406 36964253

[141] Tang M Yin S Zeng H Huang A Huang Y Hu Z. The P286R mutation of DNA polymerase epsilon activates cancer-cell-intrinsic immunity and suppresses endometrial tumorigenesis via the cGAS-STING pathway. Cell Death Dis. (2024) 15:69. doi: 10.1038/s41419-023-06418-3 38238314 PMC10796917

[142] Li MZ Wen XY Liu XQ Wang YQ Yan L. LPS-induced activation of the cGAS-STING pathway is regulated by mitochondrial dysfunction and mitochondrial DNA leakage in endometritis. J Inflammation Res. (2022) 15:5707–20. doi: 10.2147/JIR.S374318 PMC9550576 36238763

[143] Zeng X Li X Zhang Y Cao C Zhou Q. IL6 Induces mtDNA Leakage to Affect the Immune Escape of Endometrial Carcinoma via cGAS-STING. J Immunol Res. (2022) 2022:3815853. doi: 10.1155/2022/3815853 35692503 PMC9184159

[144] Kim M Mahmood M Reznik E Gammage PA. Mitochondrial DNA is a major source of driver mutations in cancer. Trends Cancer. (2022) 8:1046–59. doi: 10.1016/j.trecan.2022.08.001 PMC9671861 36041967

[145] Mok BY Kotrys AV Raguram A Huang TP Mootha VK Liu DR. CRISPR-free base editors with enhanced activity and expanded targeting scope in mitochondrial and nuclear DNA. Nat Biotechnol. (2022) 40:1378–87. doi: 10.1038/s41587-022-01256-8 PMC9463067 35379961

[146] Silva-Pinheiro P Nash PA Van Haute L Mutti CD Turner K Minczuk M. *In vivo* mitochondrial base editing via adeno-associated viral delivery to mouse post-mitotic tissue. Nat Commun. (2022) 13:750. doi: 10.1038/s41467-022-28358-w 35136065 PMC8825850

[147] Gammage PA Viscomi C Simard ML Costa ASH Gaude E Powell CA. Genome editing in mitochondria corrects a pathogenic mtDNA mutation in vivo. Nat Med. (2018) 24:1691–5. doi: 10.1038/s41591-018-0165-9 PMC6225988 30250142

[148] Bacman SR Kauppila JHK Pereira CV Nissanka N Miranda M Pinto M. MitoTALEN reduces mutant mtDNA load and restores tRNA(Ala) levels in a mouse model of heteroplasmic mtDNA mutation. Nat Med. (2018) 24:1696–700. doi: 10.1038/s41591-018-0166-8 PMC6942693 30250143

[149] King MP Attardi G. Isolation of human cell lines lacking mitochondrial DNA. Methods Enzymol. (1996) 264:304–13. doi: 10.1016/S0076-6879(96)64029-4 8965704

[150] Bayona-Bafaluy MP Manfredi G Moraes CT. A chemical enucleation method for the transfer of mitochondrial DNA to rho(o) cells. Nucleic Acids Res. (2003) 31:e98. doi: 10.1093/nar/gng100 12907750 PMC169990

[151] Divakaruni AS Paradyse A Ferrick DA Murphy AN Jastroch M. Analysis and interpretation of microplate-based oxygen consumption and pH data. Methods Enzymol. (2014) 547:309–54. doi: 10.1016/B978-0-12-801415-8.00016-3 25416364

[152] Young CKJ Young MJ. Comparison of HepaRG cells following growth in proliferative and differentiated culture conditions reveals distinct bioenergetic profiles. Cell Cycle (Georgetown Tex. (2019) 18:476–99. doi: 10.1080/15384101.2019.1578133 PMC6422474 30755072

